# Boosting the overall electrochemical water splitting performance of pentlandites through non-metallic heteroatom incorporation

**DOI:** 10.1016/j.isci.2022.105148

**Published:** 2022-09-15

**Authors:** Mohamed Barakat Zakaria Hegazy, Karim Harrath, David Tetzlaff, Mathias Smialkowski, Daniel Siegmund, Jun Li, Rui Cao, Ulf-Peter Apfel

**Affiliations:** 1 Inorganic Chemistry I, Faculty for Chemistry and Biochemistry, Ruhr University Bochum, 44801 Bochum, Germany; 2 Department of Chemistry, Faculty of Science, Tanta University, Tanta 31527, Egypt; 3 Department of Chemistry, Southern University of Science and Technology, Shenzhen 518055, China; 4 Department of Chemistry and Key Laboratory of Organic Optoelectronics & Molecular Engineering of Ministry of Education, Tsinghua University, Beijing 100084, China; 5 Fraunhofer Institute for Environmental, Safety and Energy Technology UMSICHT, 46047 Oberhausen, Germany; 6 Key Laboratory of Applied Surface and Colloid Chemistry, Ministry of Education, School of Chemistry and Chemical Engineering, Shaanxi Normal University, Xi’an 710119, China

**Keywords:** Chemistry, Inorganic chemistry, Catalysis, Electrochemical materials science, Materials science

## Abstract

We report on synthesis of the heterotrimetallic pentlandite-type material Fe_3_Co_3_Ni_3_S_8_ (FCNS) in presence of suitable phosphorus-(FCNSP) and nitrogen-(FCNSN) donors for the overall electrochemical water splitting. Throughout the experiments, a preferential incorporation of N into the FCNS-lattice is observed whereas the addition of phosphorus generally leads to metal-phosphate-FCNS composites. The obtained FCNSP, FCNSN, and FCNSNP facilitate the oxygen evolution reaction (OER) at 100 mAcm^−2^ in 1.0M KOH with overpotentials of 479, 440, and 427 mV, respectively, outperforming the benchmark IrO_2_ (564 mV) and commercial Ni metal powder (>600 mV). Likewise, FCNSN and FCNSNP reveal an improved performance toward the hydrogen evolution reaction (HER) in 0.5M H_2_SO_4,_ outperforming the pristine FCNS. All materials revealed high stability and morphological robustness during OER and HER. Notably, DFT calculation suggests that N and P doping boost the OER activity of the pristine FCNS, whereas N doping enhances the HER activity.

## Highlights

• Fine Fe_3_Co_3_Ni_3_S_8_ (FCNS) nanoparticles were prepared through ball milling methods
• Subsequent regulated thermal treatment in inert ambientes produces N- and P-doped FCNS
• The obtained materials showed high activity toward electrochemical water splitting
• DFT calculation confirms that N and P doping boosts OER, whereas N enhances HER

## Graphical abstract

**Figure:**
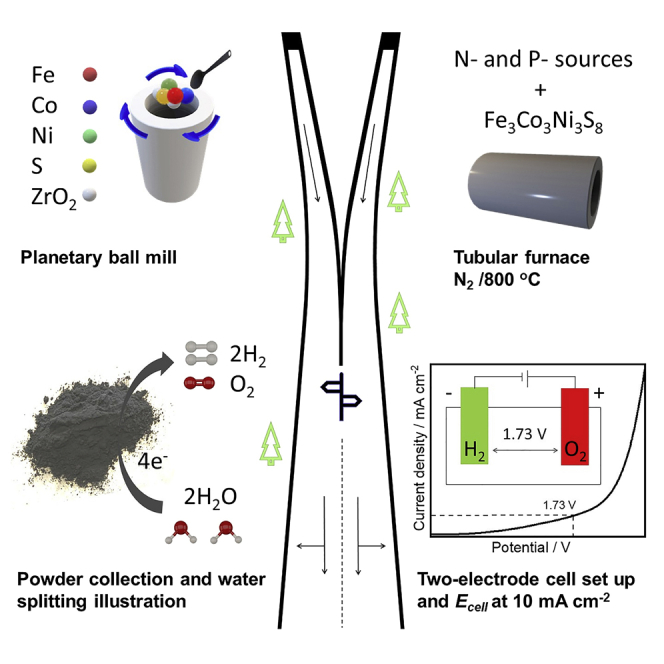

## Introduction

The race is on to design efficient and long-term stable materials for future economic and sustainable energy conversion processes (McHugh et al., 2020; Xu et al., 2020). Along this line, the global demand stimulates researchers everywhere to replace fossil fuels with clean and sustainable energy sources to fight the climate change and avoid additional environmental pollution. The desired goal is to solely exploit clean and sustainable energy sources with zero carbon emission by 2050 (Sun et al., 2021). Herein, water electrolysis emerged as key to achieve this goal (Lei et al., 2019), via the sustainable production of green hydrogen as high-density energy carrier with zero carbon emission on combustion (van Renssen, 2020). As of now, the industrial hydrogen production almost entirely stems from steam reforming of natural gases (∼96%), which is accompanied with the emission of a huge amount of carbon dioxide (CO_2_) in air (Pareek et al., 2020).

Water splitting includes the hydrogen evolution reaction (HER) (Hegazy et al., 2021) and oxygen evolution reaction (OER) (Zakaria et al., 2020b). A key issue associated with water electrolysis, however, is the amount of energy required to sustain efficient hydrogen production because of multiple electrons and protons transfer and the consequent O=O and H-H bond formation. To achieve sustainable and economic hydrogen production through water electrolysis, new materials catalyzing both the OER as well as HER with high efficiency are urgently needed to replace the scarce and costly noble metal catalysts, e.g., Ir or Ru in-proton exchange membrane (PEM) setups or to improve the performance of commonly utilized Ni-based alloys in alkaline (AEL) water electrolyzers (Li et al., 2021; Wang et al., 2021).

Along this line, transition metal-rich inorganic materials have recently emerged as vital and important alternatives to precious metal electrocatalysts because of their rich stoichiometric flexibility, high stability, achievable pseudo-metallic conductivity as well as the ease of preparation from cheap and readily available starting materials (Dou et al., 2019; Siegmund et al., 2020). Within this family of metal-rich inorganic materials, the transition metals are commonly accompanied by a variety of main group elements forming, e.g., oxides (Simon et al., 2021; Zakaria et al., 2015), sulfides (Bentley et al., 2018; Smialkowski et al., 2021; Tetzlaff et al., 2021), carbides (Zakaria et al., 2016, 2017), borides (Yao et al., 2021), phosphides (Pu et al., 2020), selenides (Desalegn et al., 2022; Feng et al., 2019; Xia et al., 2020), and double layered hydroxides (Sun et al., 2020). Despite the almost infinite number of conceivable materials, there is still no real competitor to replace current electrocatalysts in AEL and PEM technologies and achieve the efficiency and stability needed for large-scale practical applications to date (Grigoriev et al., 2020; Siegmund et al., 2021). Therefore, there is still a need to develop new materials for the overall electrochemical water splitting.

In this context, especially metal-rich sulfides were suggested to be a promising material class (Smialkowski et al., 2019; Wu et al., 2020; Xu et al., 2021; Zhang et al., 2020a). For example, the pentlandite Co_9_S_8_ showed promising behavior as a bifunctional electrocatalyst for water-splitting and enabled HER at an overpotential of 264 mV at a current density of 10 mAcm^−2^ (Tan et al., 2019). Along this line, our group developed highly conductive Fe_4.5_Ni_4.5_S_8_ pentlandite with a high potential for hydrogen production, showing an over-potential of as low as 190 mV at 10 mAcm^−2^ after its electrochemical activation (Konkena et al., 2016). The high activity was subsequently shown to arise from the defined intermetallic surface assembly and the formation of the intermediary metal hydrides with its close structural relationship to natural [FeNi]-hydrogenases (Zegkinoglou et al., 2017). Moreover, similar pentlandites were also shown to facilitate the OER, and Fe_5_Ni_4_S_8_ nanoparticles allowed OER at 200 mV overpotential to obtain a current density of 10 mAcm^−2^ (Xuan et al., 2019). Nowadays, several reports show the potential of pentlandites to act as bifunctional HER and OER catalyst under specific conditions. (Feng et al., 2015a; Huang et al., 2017; Zhu et al., 2015)

Notably, a facile and scalable synthesis procedure toward pentlandites of the type (M_1_M_2_)_9_S_8_ (where M_1_ and M_2_ are Fe and Ni, respectively) was recently shown via a direct mechanochemical approach and allowed for a simple as well as precise stoichiometric elemental control allowing the easy integration of this materials into larger-scale electrolyzers (Tetzlaff et al., 2020). Along this line, we recently reported on trimetallic pentlandites (Fe_3_Co_3_Ni_3_S_8_), which revealed promising acidic HER performance in combination with an Ir anode showing a full cell potential for the overall water splitting of just 2.6 V at a current density of 1.5 A cm^−2^ at 80 °C for an extended period (Smialkowski et al., 2022).

Despite the promising developments in the field, we opted to further improve the performance of this material class for HER and OER in alkaline medium. In addition, we aim to understand the fundamental structure/activity relationships that allow us to further improve the catalytic performance in a knowledge-guided approach.

Therefore, we herein present new synthetic protocols to dope the stable trimetallic Fe_3_Co_3_Ni_3_S_8_ pentlandite (FCNS) with non-metallic P and N and reveal the materials performance for the overall water splitting. In addition, we extend the current mechanochemistry approach to FCNS nanoparticles, thereby showing the general applicability of the synthesis route toward pentlandite materials. We showcase that heteroatom-doping improves the material’s conductivity and increases the number of active surface sites and the materials intrinsic activity, leading to an elevated performance toward the overall electrochemical water splitting in alkaline medium. The electrochemical performance of doped pentlandite-materials was investigated in alkaline and acidic solutions benchmarked with commercial IrO_2_, Ir black as well as Ni metal powders, and the pristine pentlandite.

These experiments are further supported by DFT calculations showing that nitrogen and phosphorus doping significantly tunes the binding of the intermediates on the materials surface, while at the same time weakening the ∗OH, ∗O, and ∗OOH adsorption energies compared to pristine FCNS for a better OER performance. At the same time, N doping enhances the hydrogen adsorption activity at Ni and Fe in octahedral sites leading to a significant improvement in the HER activity for the overall electrochemical water splitting.

## Results and discussion

### Synthesis & characterization

**Scheme 1:**
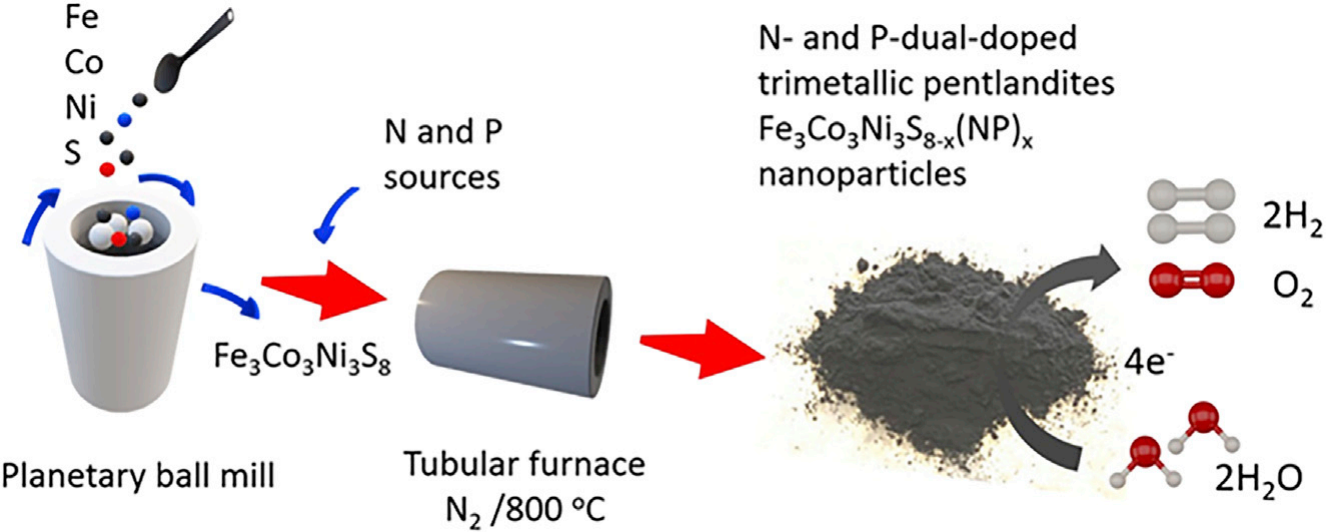
Schematic illustration for the synthetic pathway of heteroatoms-doped trimetallic FCNS pentlandites for the overall electrochemical water splitting

Contrary to our previously described method to synthesize Fe_3_Co_3_Ni_3_S_8_ (FCNS) at high temperatures in sealed ampoules under the exclusion of air, the exothermic nature of this synthesis procedure renders any thermal up-scaling attempts of this material unpractical. Therefore, we aimed to adopt our recently described ball milling pathway for bimetallic pentlandites toward FCNS materials (Tetzlaff et al., 2020). Following up on this method, the pure elements were subjected for a mechanochemical reaction under an inert atmosphere. The thus obtained FCNS material shows the same materials properties and spectroscopic characteristics as the material described before (Smialkowski et al., 2022). Thus, we clearly demonstrate the concept of ball milling is universal and can be easily transferred also to tertiary pentlandite materials. Subsequently, heteroatom-doping of pentlandite materials was performed via a downstream doping of FCNS powder materials by an annealing process at 800°C in the presence of heteroatom sources (Scheme 1).

The crystal structure and phase purity of the as-synthesized powders were examined using PXRD (Figures S1A, S1B and S2). All materials reveal dominant patterns matching the reflexes of the pentlandite (COD 9000429; PDF card no. 30–0444 (Zeng et al., 2021). After thermal treatments at 800°C it is obvious that the powder patterns intensity and reflex sharpness increased, indicating a higher degree of crystallinity. Yet, peak positions were not significantly altered, revealing overall intact pentlandite phases. However, it must be mentioned that the P-doped samples, additional foreign phases are observed which are attributed to metal phosphorus and/or phosphate impurities indicating the formation of a pentlandite/metal phosphide composite material. The analysis of foreign XRD patterns reflect peaks assignable to some extent to Ni_3_S_2_, NiS, and Ni_4.5_S_4_ in case of FCNSN sample (Figure S3), and at the same time Co_2_O_12_P_4_, NiS, and Co_3_S_4_ in case of FCNSP sample (Figure S4) during annealing. Likewise, as is visible from PXRD of the cyanuric chloride, no precursor material is found in the FCNS materials (Figure S5) indicating a complete decomposition/removal of the organic source (Figures S2 and S5). This result was further confirmed by FTIR measurements, where we could not find any peaks assignable to heteroatom organic sources (Figure S6). The additional peaks can be assigned to metal-phosphide/oxidized phosphate and metal-sulfide bonds as well as CO_2_ from the air (Baruah et al., 2019; Wood et al., 2012).

**Figure 1:**
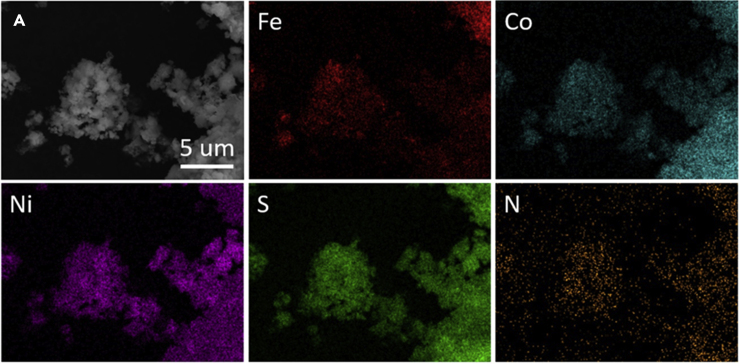
Scanning electron micrographs data of the as-prepared FCNSN sample and the correspond elemental mapping images

In addition, scanning electron microscopy (SEM) coupled with energy dispersive X-ray spectroscopy (EDX) measurements were performed to investigate the morphology and elemental distribution of all samples (Figures 1, S1C, S7, and S9). Although the data for FCNS clearly reflects the existence of solely Fe, Co, Ni, and S atoms homogeneously overlapped (Figure S7), surface atom mapping images reflect the existence of additional N in FCNSN (Figure 1) and P in FCNSP material (Figure S9). The overlapping of all elements in case of FCNSN suggests the successful doping of N into the pentlandite lattice (Figure 1). However, the P atom distribution does not reveal homogeneous overlapping with other elements, which confirms that FCNSP as well as FCNSNP are mixed pentlandite-metal phosphide composite materials (Figures S1C and S9). To estimate the particle size, we have performed high resolution SEM imaging on FCNS powder (Figure S8). It is obvious that the average particle size is in the nanoregime.

Furthermore, thermogravimetric analysis (TGA) and differential scanning calorimetry (DSC) were performed to study the thermal stability of FCNS derivatives and reflect the thermal robustness up to 880°C in nitrogen atmosphere (Figure S7B). Only thereafter, a negligible weight loss of less than 5% is observed in the TGA curve which can be attributed to a partial loss of sulfur and/or mositure. In the DSC, there is one distinct peak visible at around ∼700°C which is well-known for pentlandites (Sugaki and Kitakaze, 1998). The TGA curve of FCNSNP reveals almost typical behavior of the pristine FCNS indicating its thermal robustness as well (Figure S10). At the same time, the two bands characteristics of pentlandite phase in differential thermal analysis (DTA) disappeared after N and P incorporation indicating a change in the thermal behavior due to the change in sulfur content which is in accordance to previous reports (Figure S10) (Sugaki and Kitakaze, 1998).

Subsequently, XPS analysis of FCNS, FCNSP, FCNSN, and FCNSNP samples were performed to determine the surface composition and oxidation state of the individual elements (Figures S11–S14). Photopeaks at around 713 and 726 eV are assignable to Fe 2*p*_3/2_ and Fe 2*p*_1/2_orbitals reflecting the presence of Fe^3+^ species (Piontek et al., 2019; Wang et al., 2019). An additional photopeak at around 782 eV with a satellite photopeak at around 786 eV can be assigned to the Co 2*p*_3/2_ orbital of the Co-S bond (Xi et al., 2020). The photopeak of Ni 2*p*_3/2_ orbital was deconvoluted into one main photopeak at around 855 eV accompanied with a satellite peak at around 860 eV revealing the presence of an additional Ni^2+^ center (Hu et al., 2019; Kim et al., 2021). Likewise, the photopeak of S 2*p* orbital was deconvoluted into two peaks at around 162 and 163 eV characteristic for metal-S bond of S^2−^ species (Smialkowski et al., 2019; Zakaria et al., 2020a). These XPS results are in line with our previous report on trimetallic pentlandites. Although these XPS patterns are common in all materials, the XPS spectrum of FCNSN and FCNSNP revealed the N 1*s* spectrum with two deconvoluted peaks at 399.9 and 401.5 eV which can be assigned to metal-nitrogen bonds and graphitic nitrogens originated from amorphous carbon residue, respectively (Figures S13–S14). Likewise, FCNSP and FCNSNP samples additionally exhibit several P 2*p* photopeaks deconvoluted into three peaks at around 133.6, 134.6, and 135.7 eV, which can be assigned to the metal phosphides/phosphates (PO_x_) (Figures S12 and S14) (Yang et al., 2019).

To determine the Fe, Co, and Ni percentages, inductively coupled plasma-optical emission spectrometry (ICP-OES) analysis was performed (Table S1). Meanwhile, S and P and N atoms percentages were determined by CHNS and ion chromatography analyses, respectively (Table S1). The results confirm presence of P and N atoms by annealing.

### OER activity of the developed pentlandite materials

**Figure 2:**
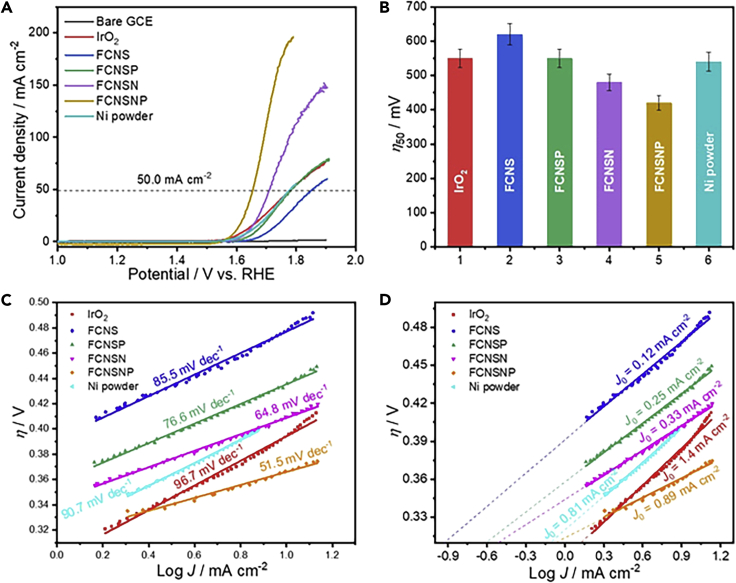
Electrocatalytic OER performance of the materials on a glassy carbon electrode (GCE) (A) LSV curves in 1.0 M KOH solution at scan rate of 5mVs^−1^, (B) the estimated overpotential at 50mAcm^−2^, (C) derived Tafel plots from LSV curves, and (D) the estimated exchange current density.

**Figure 3:**
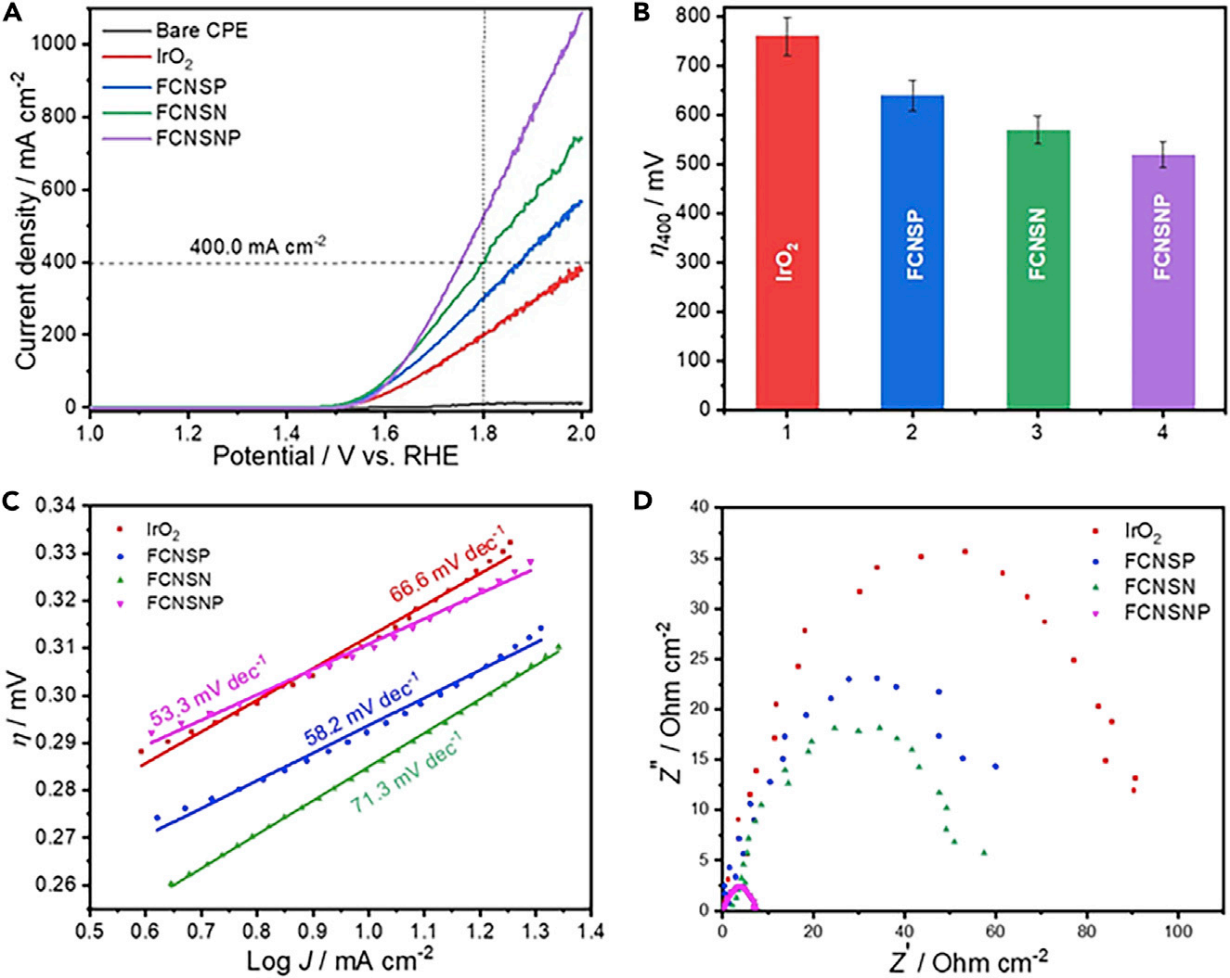
Electrocatalytic OER performance of the materials on carbon paper electrode (CPE) (A) LSV curves in 1.0 M KOH solution at scan rate of 5mVs^−1^, (B) the overpotential at 400mAcm^−2^, (C) derived Tafel plots from LSV curves, and (D) EIS Nyquist arc measurements of IrO_2_, FCNSP, FCNSN-C, and FCNSNP.

The established new materials were then applied in the electrochemical oxygen evolution reaction (OER) in alkaline solutions. The electrochemical performance of our materials toward OER was initially evaluated on glassy carbon electrode (GCE) as a working electrode in 1.0M KOH aqueous solution in comparison to the commercially available IrO_2_, Ni, and Ir black benchmark catalysts. The linear sweep voltammetry (LSV) curves highlight the OER performance of our FCNSP, FCNSN, and FCNSNP materials (Figure 2A) and overpotentials of 349 mV for FCNSNP, 390 mV for FCNSN, 419 mV for FCNSP and 421 mV for Ni metal at 10.0 mAcm^−2^ (Figures 2B and S15A) were observed. Of interest, the performance of our materials was found to be better than pure Ni and IrO_2_, as shown in Figures S15 and 2B respectively at higher current densities (50 and 150 mAcm^−2^). The low Tafel slopes of FCNSNP (44.09 mV dec^−1^) and FCNSN (62.77 mV dec^−1^) further confirm the improved OER performance of our materials over Ir and Ni metals (Figure S15B) and previously reported materials (Table S2) under basic conditions. In addition, the long-term chronoamperometry tests of FCNSNP reveal the excellent durability of FCNSNP material overIr and Ni metals (Figure S15C). The derived Tafel plots (Figure 2C) and the calculated value of slopes reflect the rapid production of oxygen in the following order: FCNSNP (51.5 mV dec^−1^) > FCNSN (64.8 mV dec^−1^) > FCNSP (76.6 mV dec^−1^) > FCNS (85.5 mV dec^−1^) > Ni (90.57 mV dec^−1^) > IrO_2_ (96.7 mV dec^−1^). In addition to the Tafel analysis, the exchange current density (*J*_0_) provides further insights into the reaction rate/overall kinetics and can be calculated from the intercept (Log *J*_0_) (Figure 2D). The high value for *J*_0_further supports the fast reaction rate and kinetics for oxygen production (Figure 3D) and shows the beneficial role of heteroatom incorporation (Hegazy et al., 2021).

Electrochemical impedance spectroscopy (EIS) measurements were then performed at a potential of 1.708 V versus RHE over the frequency range from 20 Hz to 20 kHz to determine the charge-transfer resistance (*R*_ct_) of the electrodes (Figure S16A). It is well known that pentlandites are highly conductive (Piontek et al., 2018), which is again reflected in our experiments by the small Nyquist arc and is smallest in our materials indicating the smallest *R*_ct_ value and a fast charge transfer between electrode-electrolyte interface. Moreover, FCNSNP on GCE undergoes an activation in the first 15 h and then shows a steady performance for the next 5 h as revealed by the chronoamperometry responses recorded at a fixed potential of 1.558 V versus RHE (Figure S16B). This enhancement in OER performance and stability can be explained by the electrochemical activation of the electrode during electrocatalytic OER because of formation of thermodynamically stable oxyhydroxide phases on the surface, which was intensively studied previously (Amin et al., 2021; Jin, 2017; Xu, 2019; Yang et al., 2020a).

The turnover frequency (TOF) as a direct measure of numbers of electrocatalytic active sites of the nanoparticle surface toward OER was estimated (Quast et al., 2021). The TOF for OER was calculated using the equation $TOF\;=\;\frac{j\times a}{4\times n\times f}$ where *j* is the current density at a given overpotential (424 mV), *a* is the surface area of the electrode (0.071 cm^2^ for the GCE), *4* is the number of electrons transferred in the OER, *n* is the number of moles of materials used for the OER, and *F* is Faraday’s constant (96,485Cmol^−1^). The calculated TOF at 424 mV for IrO_2_, FCNS, FCNSNP, FCNSN, and FCNSNP was 0.028 s^−1^, 0.016 s^−1^, 0.047 s^−1^, 0.075 s^−1^, and 0.287 s^−1^, respectively, reflecting the enhancement of the active sites in our materials.

To achieve higher current densities, we examined the OER performance of our materials on carbon paper electrode (CPE). Of interest, our FCNSNP, FCNSN, and FCNSP on CPE again showed an improved OER performance as compared to the benchmark commercial IrO_2_ (201 mA cm^-2^) with current densities of 537, 399, and 308 mAcm^−2^, respectively, at 1.8 V versus RHE (Figure 3A) were observed for the individual materials. Notably, bare CPE does not contribute to the overall catalytic activity (Figure 3A). Similar trends can also be observed when a constant current density of 400 mAcm^−2^ is applied to the FCNS derivatives (Figure 3B). The small Tafel slopes (Figure 3C) as well as Nyquist arc measurements (Figure 3D) reflect the fast kinetics for oxygen production and high conductivity of the prepared electrodes. Although during long-term stability tests the materials showed high stability up to a current density of 500 mAcm^−2^ (Figures S17A and S17B), complete material deactivation is observed within 5h at 1000 mAcm^−2^which, we anticipate, is due to the CPE oxidation at elevated current densities (Figure S17B). Thus, a platinum electrode (PtE) instead of the CPE was used at the same experimental conditions and comparable chronopotentiometric experiments at 1000 mAcm^−2^ as well as chronoamperometric measurements at constant potentials (1.558 V versus RHE) reveal that the observed deactivation process clearly stems from the CPE electrode support and not the material itself (Figures S17C and S17D).

It is worth to mention that the FCNSNP-PtE showed a significant OER performance with an overpotential of 398 mV at 10 mAcm^−2^ compared to a bare Pt electrode (706 mV) indicating the high OER performance originated from our material not the Pt substrate (Figure S18). This hypothesis is further supported by a control experiment performing LSV measurements using a Pt mesh or a carbon rod as counter electrode and carbon paper as a working electrode before and after *iR* compensation (Figure S19). Obviously, the overpotentials for the different experiments are comparable. In addition, it should be considered that we exclusively used H-type cells for the electrochemical measurements (Figures S20 and S21) with both anode and cathode space being separated by a membrane. Here, the counter electrode and working one are separated through a glass tube bridge with a membrane fixed in the middle to avoid any effect from the counter electrode. In addition, post analysis showed no deposition of Pt on any electrode.

The ECSA of CPE, FCNSP/CPE, FCNSN/CPE, and FCNSNP/CPE was estimated by measuring the double-layer capacitance (*C*_dl_) current calculated from the slope of the dielectric current density and the scan rate. The bare CPE shows very week activity derived from *C*_dl_ (Figure S22). The FCNSP/CPE, FCNSN/CPE, and FCNSNP/CPE showed high *C*_dl_ values of 250, 300, and 284 mFcm^−2^, respectively (Figure S23). The estimated ECSA numerical values are 15.3, 18.7 and 17.7 cm^2^, respectively (Figure S23). This result further confirms the enhancement of electrochemical active sites of the materials surface through element doping or composite formation, respectively.

To further gain insights into the improved OER performance of doped pentlandite, we investigated the OER activity of pristine FCNS, FCNSN, and FCNSP by using the computational standard hydrogen electrode model coupled with the self-consistent theoretical overpotential method (Nørskov et al., 2004; Rossmeisl et al., 2007). Because of the harsh conditions of OER, first, the structural stability for each surface at the given electrode potential was examined. As shown in Figure S24, we calculated the surface Pourbaix diagrams of each surface covered by different coverage of OH∗ and O∗. For pristine FCNS, the surface is chemisorbed by a 7/4 monolayer (ML) of OH∗ under a potential U ≈ 1.30 V versus RHE. However, as the potential increases, the surface would further oxidize to be covered by 7/4 ML of O∗. Similarly, we observe a similar trend for FCNSN and FCNSP. Specifically, the FCNSN and FCNSP oxidized by 7/4 ML of O∗ at a low electrode potential of U ≈1.15 V and U ≈1.00 V, respectively. Interestingly, it is revealed that the oxidation of the surface lead to reconstruction of the (sub)surface metal atoms with oxygen coordination, eventually leading to the formation of self-assembled amorphous metal oxide, as depicted in Figure S25. This result was further confirmed by XRD analysis of the used electrode (Figure 5B), whereas the XRD patterns reflected formation of oxide film at the surface.

**Figure 4:**
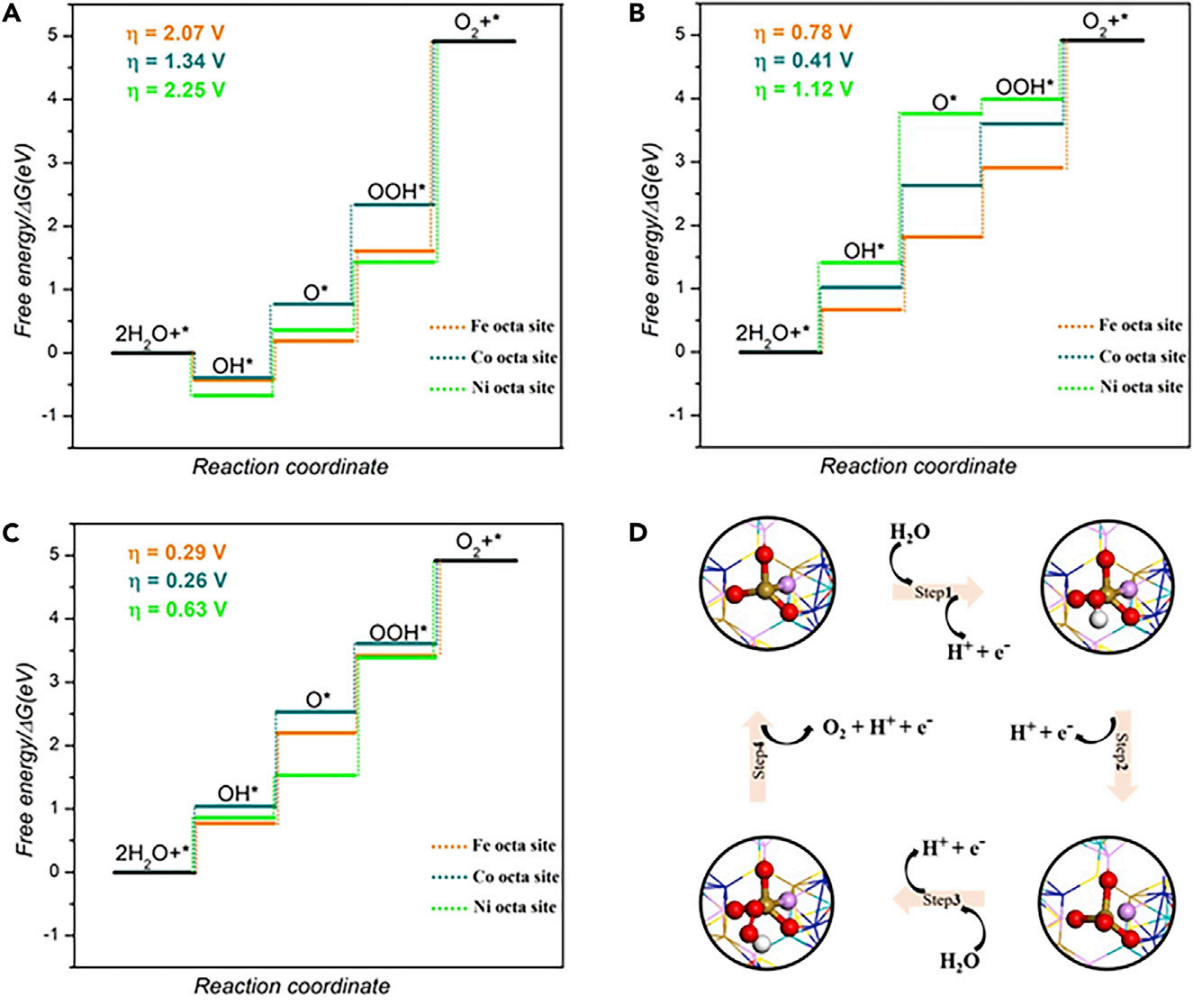
Quantum-theoretical calculations at the level of density functional theory (DFT) on the electrocatalytic OER performance Free energy diagrams of OER on Fe, Co, and Ni in (A) pristine FCNS surface, (B) FCNSN, and (C) FCNSP. (D) The computationally optimized OER intermediates on Fe site in FCNSP.

Next, we investigated the atomic-scale mechanism of OER on the three surfaces covered by 7/4 ML of O∗, as seen in Figure 4. As shown in Figure 4A, the calculated overpotential on pristine FCNS surface was 2.25, 2.07 and 1.34 V for Ni, Fe, and Co metal sites, respectively, with the oxidation of ∗OOH to O_2_ being the potential limiting step. This phenomenon can be ascribed to the strong binding between ∗OOH and metal atoms. However, as described in Figure 4B, nitrogen doping in FCNSN significantly adjusts binding of the intermediates on the surface, which weakened the ∗OH, ∗O and ∗OOH adsorption energies compared with those on pristine FCNS surface. Therefore, the calculated theoretical overpotential decreased to 1.12, 0.78 and 0.41 V on Ni, Fe, and Co metal site, respectively. On the other hand, doping of phosphorus surface leads to a dramatic decrease of the calculated theoretical overpotential to 0.63, 0.29 and 0.26 V on Ni, Fe and Co metal site (Figure 4C), respectively, explaining its good OER activity.

Of interest, we noticed that the Fe metal site (in octahedral site) on the FCNS surface, as an example, will be pulled out after the adsorption of the O∗ intermediate, whereas it will keep its geometric position in the case of FCNSP (Figure S26). Consequently, it was found a high adsorption energy of OER intermediate in the case of pristine FCNS surface as compared to those of FCNSN and FCNSP. Overall, this observation indicates that the degree of reconstruction and dynamic behavior of active site during OER reaction are strongly dependent on the composition of the surface, which eventually will affect the OER activity.

**Figure 5:**
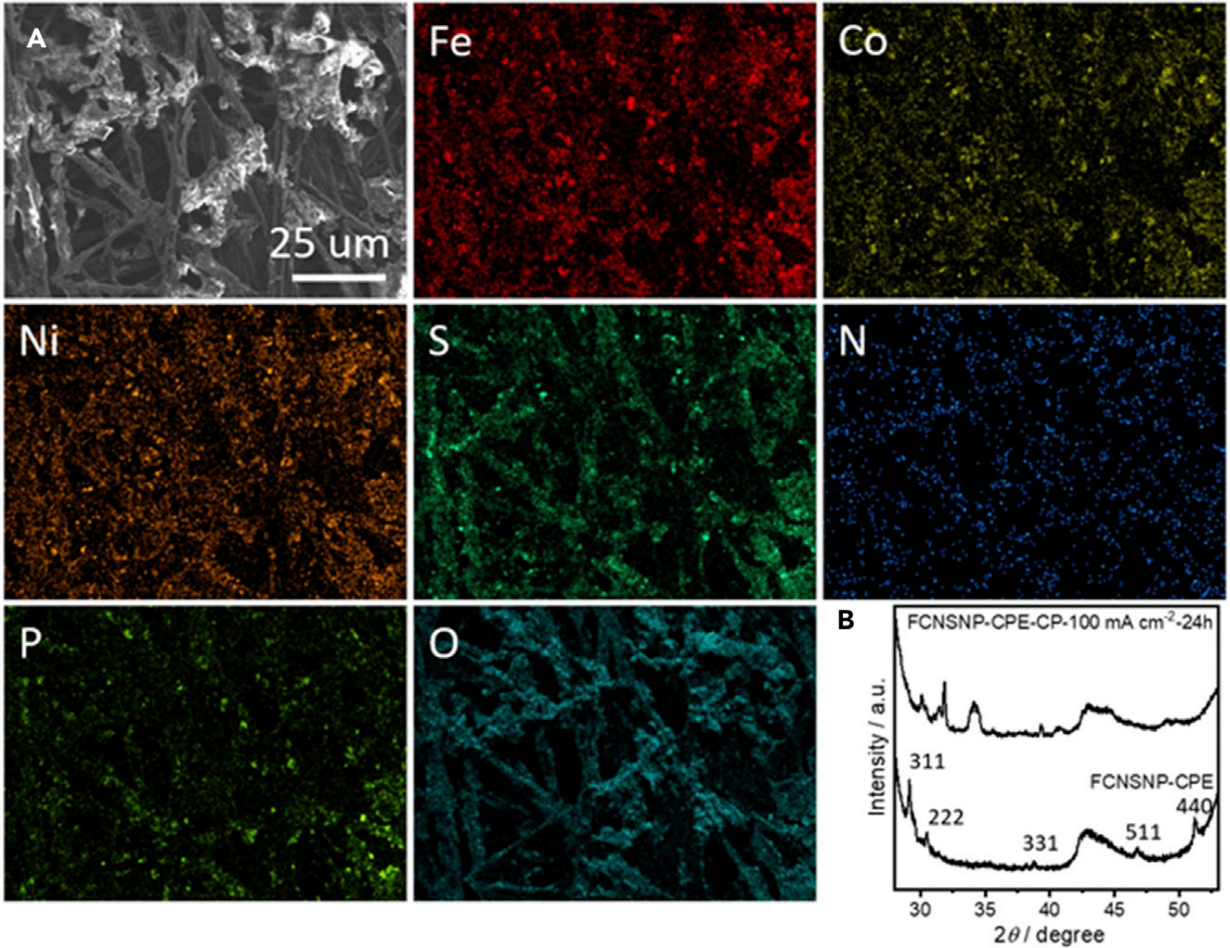
Studying morphlogy, composition, and crystal structure using scanning electron microscope attached to energy dispersive X-ray spectroscopy (SEM/EDX) and wide-angle X-ray diffraction (XRD) (A) SEM and Fe, Co, Ni, S, N, P, and O atoms mapping images of FCNSNP on CPE after chronopotentiometry test for 24 h at 100mAcm^−2^. (B) XRD patterns of FCNSNP on CPE after chronopotentiometry test for 24 h at 100mAcm^−2^.

The calculations obviously suggest a change of the surface composition. Thus, we examined the morphology and structure features of FCNSNP/CPE after chronopotentiometric runs for 24 h at 100 mAcm^−2^ (Figure 5). It is obvious that the FCNSNP composite particles are distributed over CPE (Figure 5A). Most importantly, powder XRD patterns reflect the formation the metal oxide shell because of surface oxidation during OER (Figure 5B) as was forecasted by DFT calculations in this work (Figure S25). Thus, the studies show that pentlandites readily convert into the thermodynamically more stable oxide and/or oxyhydroxide phases during OER which contribute to the high activity of the materials (Amin et al., 2021).

To check the effect of CPE on the materials composition and structure, we performed wide angle XRD analysis of the as-prepared materials on the CPE. It is obvious that all electrodes gave similar XRD patterns of pentlandite phase (Figure S27). This result further excludes the contribution of CPE in OER activity and as well as reflects chemical stability of our materials. Although the herein presented materials reveal a promising performance for OER under basic conditions, it must be noted that rapid loss in activity is observed under acidic conditions concomitant with material dissolution. Thus, these pentlandites are not suitable to be used in PEM electrolysis.

### HER activity of the developed pentlandite materials

Inspired by the composition and the hydrogenase like structure, pentlandites showed a promising performance for HER catalysis (Konkena et al., 2016). The metal rich redox centers, their tunable molecular compositions, enhanced electrocatalytic activity as well as high ionic conductivity put them in a unique position of HER catalysis (Piontek et al., 2018; Smialkowski et al., 2022). The initial evaluation of our materials for HER was performed in 1.0M KOH aqueous solution on GCE (Figure S28). The FCNSN sample showed the best performance with an overpotential of 367 mV outperforming the benchmark FCNS (587 mV) at 10 mAcm^−2^ (Figure S28A). In contrary, the FCNSNP shows an overall reduced HER performance compared to FCNSN samples with an overpotential of 525 mV at 10 mAcm^−2^. Yet, the FCNSNP HER performance improves at higher current density. In terms of Tafel slope the FCNSNP shows the best kinetics (133 mV dec^−1^) compared to FCNSN (187 mV dec^−1^) and FCNS (145 mV dec^−1^) (Figure S28B). In addition, the EIS Nyquist arc measurements give small arcs in case of doped samples revealing the high conductivity of our materials and fast charge transfer between electrode-electrolyte interface compared to pristine FCNS (Figure S28C). This enhancement might be attributed to the difference in electronegativity and synergistic effect between various components.

**Figure 6:**
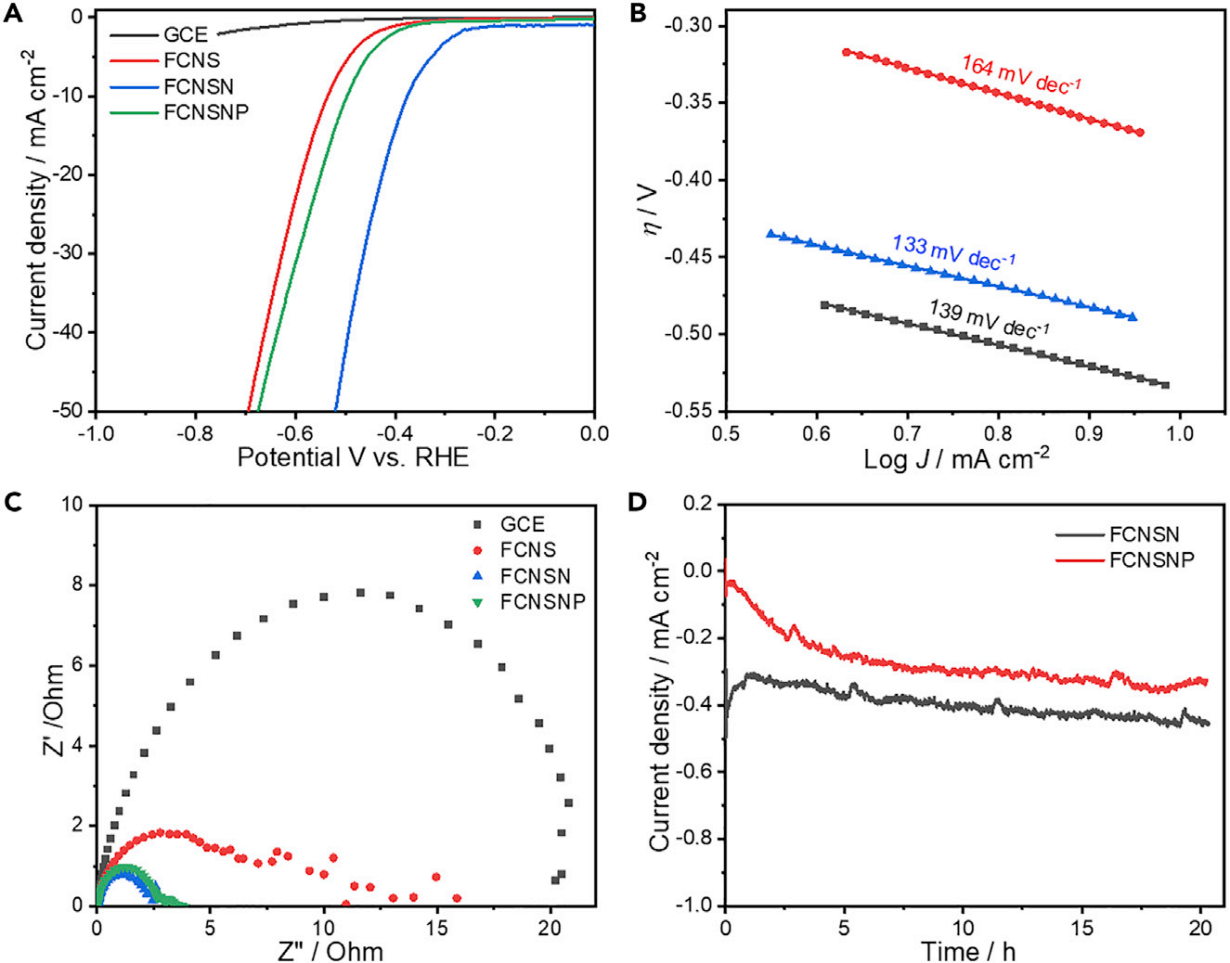
Electrocatalytic HER performance of our materials on glassy carbon electrode (GCE) (A) LSV curves of GC, FCNS, FCNSN, and FCNSNP electrodes in 0.5M H_2_SO_4_ solution at scan rate of 5mVs^−1^, (B) derived Tafel plots from LSV curves, (C) EIS Nyquist measurements on GCE electrode, and (D) chronoamperometry test for 20 h of FCNSN, and FCNSNP electrodes at −0.355 V versus RHE.

Although the activity of the doped material is improved in alkaline medium, we were eager to see their HER performance in the acidic medium (0.5M H_2_SO_4_) shown in Figure 6. As anticipated, the HER performance in acid medium improved for all materials. The FCNSN sample showed an overpotential of 344 mV at 10 mAcm^−2^ as compared to FCNS (517 mV) at 10 mAcm^−2^ and FCNSNP as well (473 mV) (Figure 6A). The kinetics of hydrogen production improved as indicated by the lower Tafel slopes of 139, 164, and 133 mV dec^−1^ for FCNS, FCNSN, and FCNSNP electrodes, respectively (Figure 6B). In addition, the FCNSN and FCNSNP showed the smallest Nyquist arc revealing the improved charge transfer between electrode-electrolyte interface (Figure 6C). Of interest, the doped materials and composites showed a good stability for long runs in acid solutions, which was confirmed by chronoamperometry test at −0.355 V versus RHE for 20 h (Figure 6D). It is obvious that the electrodes are activated at the first few hours and then showed quite stable and durable performance. This result was further confirmed through testing the FCNSN electrode performance by LSV before and after chronoamperometry at −0.355 V versus RHE for 20 h in 0.5 M H_2_SO_4_ on GCE (Figure S29). The electrode reveals significant activation and robustness, revealing an overpotential of 666 mV at a current density of 100 mAcm^−2,^ outperforming the benchmarked as-prepared FCNS (716 mV).

**Figure 7:**
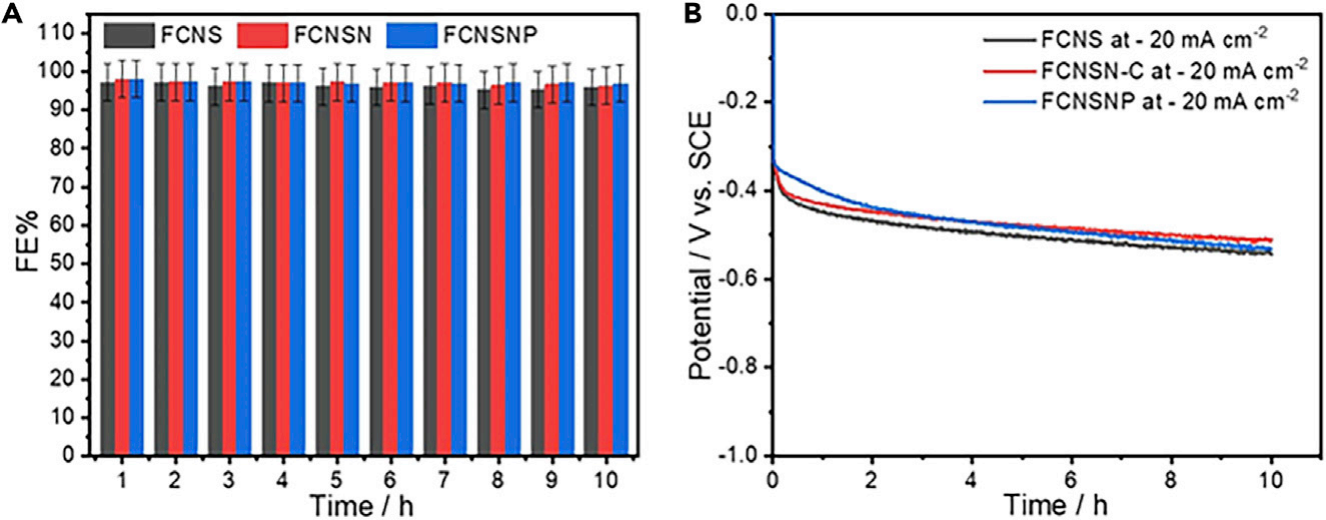
Faradic efficiency percentage (FE%) calculations using gas chromatpgraphy (GC) (A) The estimated FE% for hydrogen production from CG curves and (B) chronopotentiometry test of FCNS, FCNSN, and FCNSNP electrodes in Ar at - 20mAcm^−2^ for 10 h.

The faradaic efficiency percentage (FE%) of hydrogen production was determined within 10 h electrolysis using online gas chromatography (Figure S30). The chronopotentiometry test was performed at 20 mAcm^−2^ to determine O_2_ (Figure S31A) and at -20 mA cm^-2^ to determine H_2_ (Figure S31B) respectively. The obtained chromatograms highlight the successful OER and HER at both anode and cathode for the overall water splitting with overall achieved 98%±5faradaic efficiency for hydrogen production (Figure 7A). It is obvious that the electrodes show high stability with almost constant performance and FE% (Figures 7A and 7B). To elucidate the good HER performance of our materials, we compared it with the previously reported pentlandite materials as shown in Table S3.

**Figure 8:**
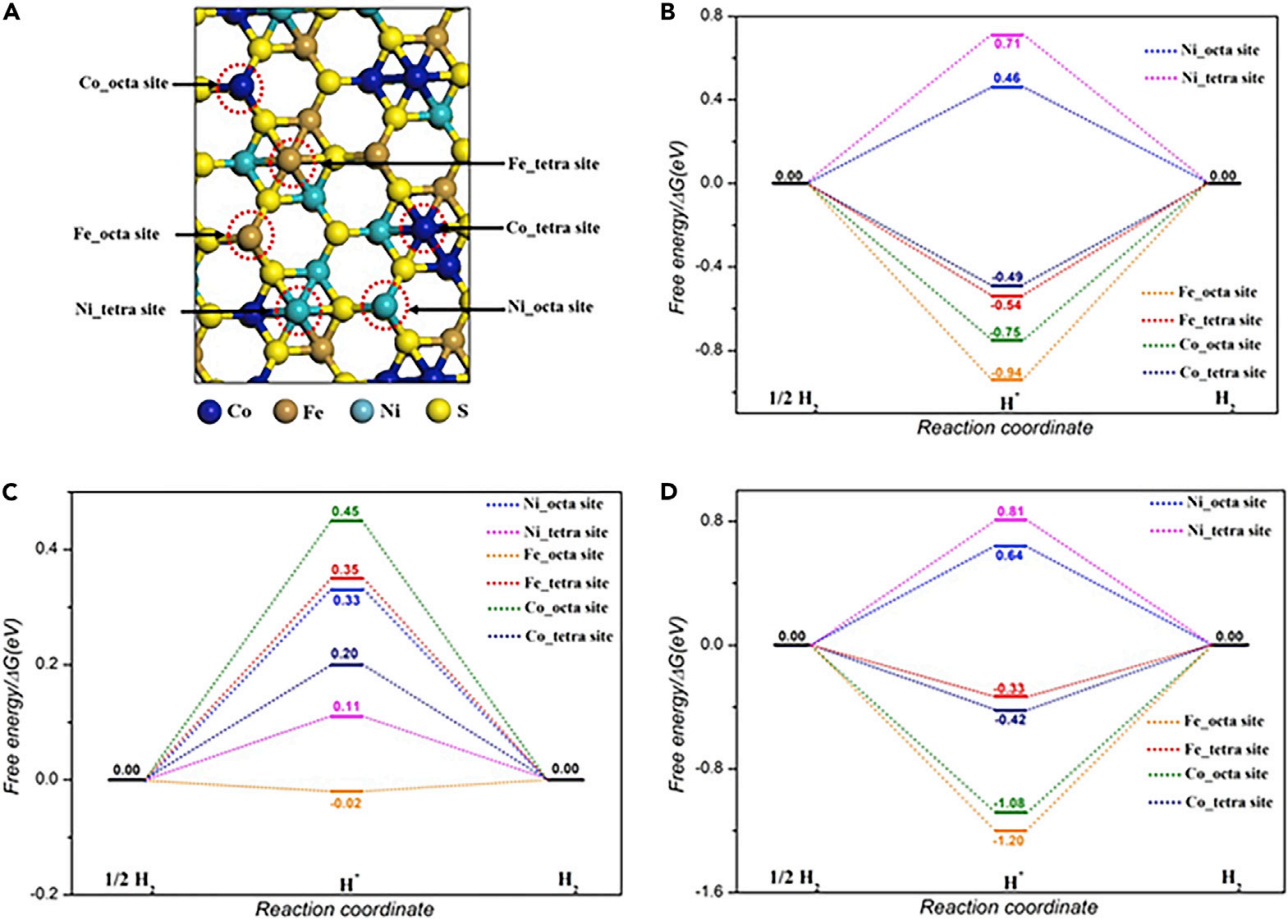
Quantum-theoretical calculations at the level of density functional theory (DFT) on the electrocatalytic HER performance (A) Top view of optimized (111) pristine FCNS surface. Free energy diagrams of HER on Fe, Co, and Ni sites in (B) pristine FCNS, (C) FCNSN, and (D) FCNSP on (111) surface.

To further understand the HER activity and the nature of active sites on the hypothetical doped FCNS, FCNSN, and FCNSP, the adsorption free energy of hydrogen for different sites were calculated to estimate their catalytic activity toward HER (Figures 8A and 8B). The hydrogen adsorption free energy is calculated at a potential U = 0 relative to the standard hydrogen electrode at pH = 0. The ΔGH∗ illustration in Figure 8B shows that the Ni metal in octahedral site and Fe and Co metals in tetrahedral site exhibit smaller ΔGH∗ (0.46eV, 0.49eV and 0.54eV respectively), indicating their superior electrocatalytic HER performance than other metals sites. Compared with pristine FCNS, nitrogen doping in FCNSN enhances the hydrogen adsorption activity of all sites. We found the global minimum energy of hydrogen adsorption on each type of metal site tends to be much closer to zero, as depicted in Figure 8C. Specifically, the improvement of H adsorption activity over the sites can be clearly observed over the Ni and Fe in octahedral sites. For example, ΔGH∗ of the Ni and Fe in the FCNSN surface has a much smaller value of 0.11eV and −0.02 than the value of 0.46eV and −0.94 eV, respectively, obtained from the identical sites on the pristine FCNS surface. In contrast, the phosphorus doping in the FCNSP surface exhibits a higher ΔGH∗ value, indicating a drop in HER performance, which is in line with the experimentally HER observation.

Next, we evaluated the possibility of anions contribution to HER activity as an active site, as illustrated in Figure S32A. Among three types of anion sites, phosphorus exhibits the smallest ΔGH∗ of −0.02 eV, highlighting the role of phosphorus as the principal active site for HER. This finding is consistent with recent outcomes that include the phosphides material (Caban-Acevedo et al., 2015; Fu et al., 2020; Kibsgaard and Jaramillo, 2014; Luo et al., 2017; Yan et al., 2018). To gain an in-depth understanding of anion dopants effect on active sites electronic structure, the projected density of states (PDOS) of the Ni metal in the octahedral site were plotted (Figure S32B). The PDOS plot shows that the N dopant significantly shifts the d band center near the E_F_ value, increasing the interaction between H∗ and Ni sites, which is beneficial to increase its HER activity. As shown in Figure S32B, the Bader charge for the Ni octahedral site from nitrogen doping surface is 0.45e, which is considerably larger than the value of 0.20e obtained from the Ni on sulfur surface, indicating electron transfer from Ni to the adjacent nitrogen atoms. Correspondingly, the interaction between Ni and H atom would enhance, and the value of ΔGH∗ decrease to be much closer to zero. An inverse trend is observed by comparing Ni octahedral site electronic structure from phosphorus doping surface with Ni site from nitrogen doping surface, explaining the drop in HER activity.

**Figure 9:**
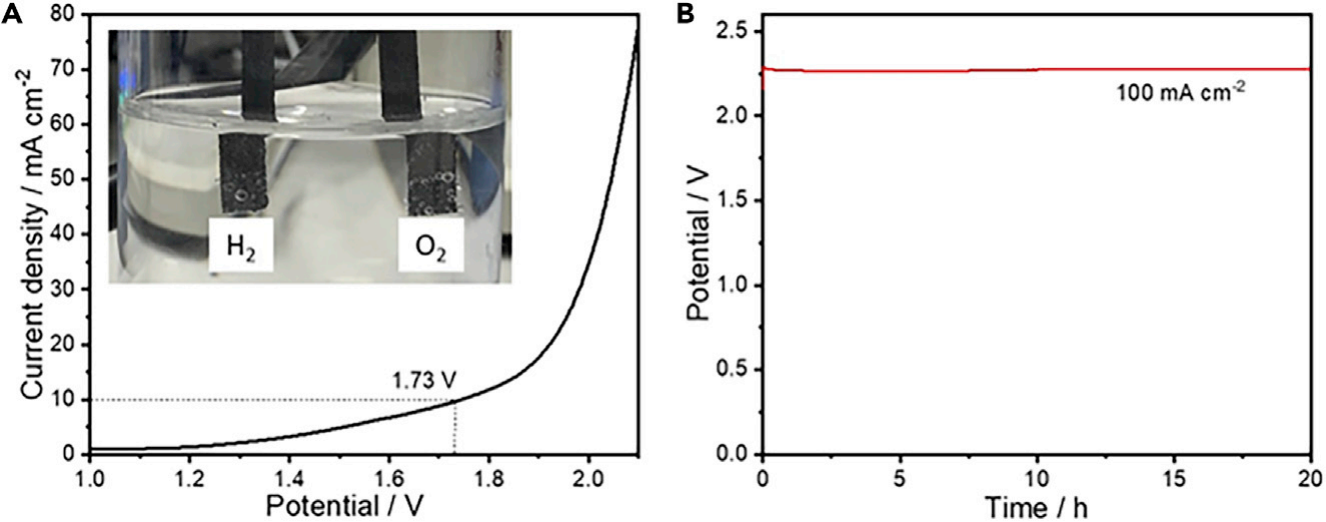
Two-electrode cell for studying the overall electrochemical water splitting using FCNSN in both anode and cathode sides (A) LSV curves for the overall water splitting using FCNSN at both cathode and anode at a scan rate of 10mVs^−1^ in 1.0 M KOH solution. (B) Chronopotentiometry test of the cell at 100mAcm^−2^.

All the aforementioned results here indicate that our materials are active and stable bifunctional electrocatalysts for OER in alkaline solutions and HER in both alkaline and acidic solutions. Therefore, to investigate the catalytic activity for the overall water splitting, we assembled the two-electrode setup using FCNSN in both cathode and anode sides in alkaline solution (inset: Figure 9A). The FCNSN electrode exhibits high activity achieving a water-splitting current density of 10 mAcm^−2^ at 1.73 V which is favorably comparable to the previously published electrocatalysts for the overall water splitting (Feng et al., 2015b; Jin et al., 2016; Xu et al., 2017). The chronopotentiometry test reveals a stable overall cell potential of around 2.276 V for 20 h at 100 mAcm^−2^, reflecting the potential application of FCNSN for overall water splitting in alkaline solution (Figure 9B). We have attached two supplementary videos showing our two-electrode set up using FCNSN in both cathode and anode sides and its performance in the start (Video S1) and after 24 h (Video S2) of the overall electrochemical water splitting at 100 mAcm^−2^.

Video S1. Shows the chronopotentiometry test at 100 mA cm-2 of the two-electrode cell using FCNSN in both anode and cathode side in the first 30 sec, related to Figure 9b

Video S2. Shows the chronopotentiometry test at 100 mA cm-2 of the two-electrode cell using FCNSN in both anode and cathode side after 24 h of continuous performance, related to Figure 9b

### Limitations of the study

Because doping of materials was carried out at elevated temperatures in inert atmosphere, precautions for safety should be taken care and the described regulated annealing procedures in the STAR Methods section should be followed carefully – a direct up scaling of the material synthesis is thus not advised. Notably, any oxygen or air flow inside the tube will lead to materials oxidations. Likewise, the amount of hydrogen and oxygen gases produced during water splitting tests is high. Thus, large carbon cloths substrate or closed cell need to be avoided or other precautions to avoid significant buildup of H_2_/O_2_ mixtures must be taken. Likewise, the same care should be applied while doing online GC measurements – the carrier gas type and pressure and injection pressure should be selected and done carefully according to the experimental description in STAR Methods section.

### Conclusion

Doping pristine FCNS trimetallic pentlandites with non-metallic heteroatoms of N (FCNSN) and P (FCNSP) was performed through a combinatorial approach involving mechanochemical methods and consequent controlled annealing processes in an inert atmosphere. In the first stage, FCNS trimetallic pentlandite-type nanoparticles was prepared by ball milling method using elemental Fe, Co, Ni, and S. In the second stage, doping of N and P atoms was carried out by regulated thermal treatments at 800°C in nitrogen gas in the presence of suitable N and P precursors. Notably, the obtained pentlandite derivates show enhanced OER performance and durability in alkaline solution and, at the same time, improved HER performance in acid solution for the overall electrochemical water splitting. The OER performance of our materials outperformed the herein used benchmark materials of pristine FCNS and commercial IrO_2_, Ir black, and Ni metal powders used in industry. DFT calculations on (111) surface of pristine FCNS, FCNSN, and FCNSP materials were further performed to gain more insights in the OER and HER behavior of N/P-modified FCNS-surfaces. Here, doping of phosphorus leads to a decrease of the calculated theoretical overpotential on Ni, Fe, and Co metal sites confriming improved OER. At the same time, N doping in FCNSN samples enhances the hydrogen adsorption activity of all metal sites for better HER. Specifically, the improvement of H adsorption activity over the sites can be clearly observed over the Ni and Fe in octahedral sites. In contrast, the phosphorus doping in FCNSP surface exhibits a higher ΔGH∗ value, indicating a drop in HER performance. In summary, we herein demonstrate that pentlandites and their N-derivatives as well as P-composites allow sophisticated HER performance under acidic conditions but decompose under acidic OER conditions. Contrary under basic conditions, the materials are promising catalysts for HER as well as OER and can be operated at high current.

## STAR★Methods

### Key resources table

**Table:** 

REAGENT or RESOURCE	SOURCE	IDENTIFIER
**Chemicals, peptides, and recombinant proteins**		

Nickel powder (99.9%, 3N)	Merck, Germany	CAS no. 7440-02-0
Iron powder (≥99%, reduced)	Merck, Germany	CAS no. 7439-89-6
Cobalt powder (99.8% trace metal basis)	Merck, Germany	CAS no. 7440-48-4
Sulfur powder (99.5–100.5%)	Merck, Germany	CAS no. 7704-34-9
Cyanuric chloride (99%)	Merck, Germany	CAS no. 108-77-0
Phytic acid solution (50% (w/w) in H_2_O)	Merck, Germany	CAS no. 83-86-3
Nafion perfluorinated resin 5% in alcohol	Merck, Germany	CAS no. 31175-20-9
Potassium hydroxide	Merck, Germany	CAS no. 1310-58-3
Sulfuric acid	Merck, Germany	CAS no. 7664-93-9
0.05 μm polishing alumina	ALS Co., Ltd, Japan	cat. no. 012620
1.0 μm polishing diamond	ALS Co., Ltd, Japan	cat. no. 012621

**Software and algorithms**		

Origin 8	Origin Lab	https://www.originlab.com/
Gamry Echem	Gamry Instruments	https://www.gamry.com/
the Vienna ab initio simulation package (VASP)	VASP Software GmbH	https://www.vasp.at/

### Resource availability

#### Lead contact

Further information and requests for resources and reagents should be directed to and will be fulfilled by the lead contact, Ulf-Peter Apfel (ulf.apfel@rub.de).

#### Material availability

Materials are available up on request.

### Method details

#### Synthesis of Fe_3_Co_3_Ni_3_S_8_(FCNS)

Synthesis of FCNS was performed via a mechanochemical process recently published by our group (Tetzlaff et al., 2020). A reaction mixture (m_total_ = 25 g) composed of stoichiometric amounts of the elements iron, cobalt, nickel, and sulfur powders were milled employing a Fritsch Pulverisette 7, premium line with ZrO_2_ milling containers (V = 80 mL) and ZrO_2_ milling balls (100 g, d = 5 mm). The reaction mixture was prepared inside a glove box to assure an inert argon atmosphere inside the milling vessel. Ball milling was performed at a constant rotation speed of 900 rpm for 4 × 60 min, with a 60 min break after each cycle.

#### Synthesis of Fe_3_Co_3_Ni_3_S_8-x_P_x_(FCNSP)

The FCNSP was prepared through a regulated annealing process of FCNS at 800°C in nitrogen gas (flow rate of 80mLmin^−1^) in the presence of phytic acid as a phosphide source (Yang et al., 2020b; Zhang et al., 2020b). FCNS powder (0.25 g) was placed in a quartz-boat, and phytic acid liquid (1.5 g) was placed in a separate quartz-boat which were both fixed in a tubular furnace with quartz tubing purged with N_2_ gas. After continuous annealing at 800°C for 3h at a rate of 2Cmin^−1^. The powder was then cooled inside the furnace at a cooling speed of 10°Cmin^−1^.

#### Synthesis of Fe_3_Co_3_Ni_3_S_8-x_N_x_(FCNSN)

Nitrogen was doped through annealing of FCNS at 800°C in an inert atmosphere in nitrogen at a flow rate of 80mLmin^−1^ in the presence of cyanuric chloride as N sources. FCNS/cyanuric chloride (wt/wt = 1/5) were mixed, grinded, and placed in a quartz-boat fixed in a tubular furnace with quartz tubing purged with N_2_ gas. The annealing process was initiated by heating from room temperature up to 300°C min^−1^ for 1 h. After continuous annealing at 300°C min^−1^ for 40 min, the temperature was raised again up to 800°C during 1 h. After further continuous annealing at 800°C min^−1^ for 2 h, the powder was cooled inside the furnace at a rate of 10°Cmin^−1^. The obtained FCNSN powder was collected and used as obtained for further testing.

#### Synthesis of Fe_3_Co_3_Ni_3_S_8-x_(NP)_x_(FCNSNP)

For the preparation of N and P dual-doped-FCNS, phytic acid liquid was used as a source of P and cyanurchloric as a source of N during annealing process in nitrogen. FCNS/cyanuric chloride (wt/wt = 1/5) were mixed, grinded, and placed in a quartz-boat fixed in a tubular furnace with quartz tubing purged N_2_ gas. Phytic acid liquid was placed in a separate quartz-boat and fixed in the same tubular furnace with quartz tubing purged N_2_ gas. The annealing process was initiated by heating from room temperature up to 300°C min^−1^ during 1 h. After continuous annealing at 300°C min^−1^ for 1 h, the temperature was raised again up to 800°C during 1 h. After further continuous annealing at 800°C min^−1^ for 2 h, the powder was cooled inside the furnace at a rate of 10°Cmin^−1^. The thermal treatment process was performed in nitrogen with a flow rate of 80mLmin^−1^.

#### Electrochemical measurements

A conventional H-type three-electrode electrochemical cell was used for testing OER and HER. The entire electrochemical tests were performed utilizing a GAMRY 1010B potentiostat. A glassy carbon electrode (GCE) with a geometric surface area of 0.071 cm^2^ and a carbon paper electrode (CPE) with a geometric surface area of 0.16 cm^2^ coated with the catalysts were used as working electrodes, saturated calomel electrode (SCE) as a reference electrode, and a platinum mesh as a counter electrode. The reported potential versus the reversible hydrogen electrode (RHE) was calculated using the following equation: $E_{RHE}\;=\;E_{SCE}\;+0.241\;+0.059\;pH$. To prepare the ink used for the working electrode, 5 mg of the catalyst was well-dispersed through sonication in a mixture of water and ethanol (950 μL, 3:1 *v/v*) until establishing a homogeneous solution. 50 μL of 5 wt % Nafion perfluorinated resin solution was poured into the prepared suspension while keeping continuous sonication for 1 h more until affording a homogeneous ink. Finally, 10 μL of the prepared ink were drop-casted onto the surface of the CPE and dried at room temperature. Likewise, 5 μL of the prepared ink were drop-casted onto the surface of GCE in case. Linear sweep voltammetry (LSV) measurements were performed in a potential range between 1.0 V and 2.0 V versus RHE for OER test and 0.2 V and −0.8 V versus RHE for HER test with a scan rate of 50mVs^−1^ in 1.0M KOH as well as 0.5 M H_2_SO_4_ solutions. Tafel plots were derived from LSV curves around the onset potential region. Cyclic voltammetry (CV) measurements were performed at different scan rates to estimate the electrochemical active surface area (ECSA). The electrochemical impedance spectroscopy (EIS) to explore charge transfer speed in both OER and HER were also investigated. The calculation of ECSA was performed according to the formula, $ECSA\;=\;R_{f}S$, in which *S* represents the real surface area of the smooth electrode, equivalent to the geometric area of the working electrode. The roughness factor (*R*_*f*_) was obtained from the formula, $R_{f}\;=\;\frac{C_{dl}}{C_{s}}$, in which the double layer capacitance (*C*_*dl*_) was equal to the slope of the double layer charging current versus the scan rate slope using this formula $i\;=vC_{dl}$. The *C*_*s*_ (general specific capacitance) corresponded to the average double layer capacitance of a smooth surface of about 20–40μFcm^−2^ (Mohd Najib et al., 2020). Finally, chronoamperometry and chronopotentiometry tests were performed for 24 h to determine the catalyst durability and stability for long-term OER and HER performance.

#### Characterizations

Powder X-ray diffraction (PXRD) measurements were performed on a Bruker D2 Phaser diffractometer equipped with a LynxEye XE-T detector operating at 30 kV acceleration voltage and 10 mA emission current using Cu K-α radiation (λ = 1.54184 Å). The data was recorded in a range from 10 to 70° 2θ. The thermogravimetric analysis (TGA) and differential scanning calorimetry (DSC) were performed using a Netzsch STA 449 F3 Jupiter equipped with nitrogen-purged SiC-Oven (<1550°C). The device was properly calibrated using In, Zn, Al, Ag, and Au. The FTIR spectra were collected using Shimadzu IR Tracer-100 with a Pike miracle ATR unit. Scanning electron microscopy (SEM) was performed on a ZEISS Gemini2 Merlin HR-FESEM equipped with an OXFORD AZtecEnergy X-ray microanalysis system for energy dispersive X-ray spectroscopy (EDX). The SEM images were recorded at an acceleration voltage of 4 kV, whereas EDX mappings were performed from 0-20 kV. XPS measurements were carried out in an ultra-high-vacuum (UHV) setup equipped with a polychromatic Al Kα X-ray source (1486.6 eV) or Mg Kα X-ray source (1253.6 eV) and a hemispherical analyzer (type CLAM2, VG, Scientific, Thermo Fisher Scientific). The base pressure in the measurement chamber was maintained at about 10^−9^ mbar. All spectra were recorded with a pass energy of 100eVat a beam current of 13 mA and a high voltage of 14 kV, which equals a Power of 13∗14 = 182 W. ICP was performed to measure the elements Fe, Ni, and Co after microwave digestion on an AnalytikJena Model ContrAA800 AAS. Nitrogen was determined with a CHNS analyzer from the company Elementar Model Vario MicroCube. Phosphorus and sulfur were measured after high-temperature pressure digestion on a Metrohm Model 883 Plus ion chromatograph.

#### Online gas chromatography (GC) measurements

Agilent 7820A GC System for gas analytics was used for qualitative and quantitative analysis of O_2_ and H_2_ gases and for faradaic efficiency percentage (FE%) calculations. The gas chromatography system is equipped with two columns (haysepq and molesieve 5A), a flame ionization detector (FID), and a thermal conductivity detector (TCD). Argon was used as carrier gas for all measurements.

#### Computational methods

The spin-unpolarized DFT calculations with the Perdew-Burke-Ernzerhof (PBE) exchange-correlation functional were performed using the Vienna ab initio simulation package (VASP) (Perdew et al., 1996). The projector augmented wave method (PAW) (Blöchl, 1994; Kresse and Joubert, 1999), with a plane-wave kinetic energy cutoff of 400 eV, was used, with the Gaussian smearing of 0.05 eV. The Brillouin zone was sampled by only the 3 × 3×1 K-point. The same k-points mesh was used for DOS calculations. Based on XRD results, the pentlandite Fe_3_Co_3_Ni_3_S_8_ model we adopted for calculation is a (111) surface, which we modeled by a (2 × 2) supercell, and a vacuum layer of 15 Å was added to eliminate artificial interactions between periodic images for modeling surface chemistry. We substituted the surface sulfur atoms with nitrogen or phosphorus atoms for doped pentlandite systems to evaluate the general trends of dopant effects. All atoms were allowed to relax during geometry optimization, and the atomic positions were optimized until the forces were less than 0.02 eV/Å. The effects of van der Waals corrections were modeled using Grimme’s method, with Becke–Johnson damping (Grimme et al., 2010). The adsorption energy of hydrogen atom ($E_{ad}^{H}$) is defined as the energy difference before and after the adsorption with respect to the gas-phase H_2_ molecule as shown in the following equation: $E_{ad}^{H}\;=\;E_{total}\;-\;E_{surafce}\;-\;1/2E\left(H_{2}\right)\;$, where $E_{ad}^{H}$, $E\left(H_{2}\right)$, and $E_{total}\;$ are the energies for the clean surface, H_2_ molecule in the gas phase, and hydrogen atom adsorbed on the surface, respectively. The Gibbs-free energy of H adsorption (ΔGH) is obtained by applying the entropy correction as shown in the equation $\Delta G_{H}\;=\;\Delta E_{H}+\;\Delta E_{ZPE}\;-\;\Delta S_{H}$. The OER performance of pure and doped Pentlandites systems can be predicted using the Gibbs-free-energy (ΔG) profiles for the following sequence of elementary OER sub-steps, according to Norskov et al. (Man et al., 2011):

$$\;\text{Step}\;\text{I}:{\text{OH}}^{-}+*\to *\text{OH}+{\text{e}}^{-}$$

$$\;\text{Step}\;\text{II}:*\text{OH}+{\text{OH}}^{-}\to *\text{O}+{\text{H}}_{2}\text{O}+{\text{e}}^{-}$$

$$\;\text{Step}\;\text{III}:*\text{O}+{\text{OH}}^{-}\to *\text{OOH}+{\text{e}}^{-}$$

$$\;\text{Step}\;\text{IV}:*\text{OOH}\to *+{\text{O}}_{2}+{\text{e}}^{-}$$

where ∗ represents the bare site and ∗OH, ∗O, ∗O_2,_ and ∗OOH denote the surface featuring different chemisorbed species. The free energy difference for all of the elementary steps above (ΔGOH∗, ΔGO∗, ΔGOOH∗) involve an electron transfer is calculated by the equation ΔG=ΔE+ΔZPE−TΔS+ΔGU+ΔGpH, where ΔE, ΔZPE, and ΔS correspond to the energy difference between adsorption energy, zero-point energy, and entropy, respectively. The adsorption energies ΔE were measured by using DFT. The ΔZPE and TΔS values were obtained from harmonic vibrational frequency calculations and DFT. ΔGU = −eU, where U represents a potential based on a standard hydrogen electrode. ΔGpH represents the Gibbs-free energy correction of the pH, noting that we consider pH = 0 in our computational investigation.

Under ideal conditions, the OER reaction with a total energy change of 4.92 eV can be driven at 1.23 V, whereas the free energy of each elementary reaction would be equally divided into 1.23 eV. Thus, the overpotential *η* is introduced to represent the additional required potential and rationalize the catalytic performance of the catalyst, which is defined in theoretical calculations as: *η* = max(ΔG_(1,2,3,4)_)/e − 1.23 eV.

## Data Availability

Data reported in this article will be shared by the lead contact on request. There is no dataset or code associated with this work. SEM, TEM, XRD, TGA, DSC, electrochemical data, and spectroscopic and chromatographic data.
