# Supplementary material for: Boosting the overall electrochemical water splitting performance of pentlandites through non-metallic heteroatom incorporation
Source: iScience. 2022 Sep 15;25(10):105148. doi: 10.1016/j.isci.2022.105148 (PMC9529978; doi:10.1016/j.isci.2022.105148)
Supplement: Document S1. Figures S1–S32 and Table S1–S3 [file mmc1.pdf]

iScience, Volume *25*

Supplemental information

Boosting the overall electrochemical water splitting performance of pentlandites through non-metallic heteroatom incorporation

Mohamed Barakat Zakaria Hegazy, Karim Harrath, David Tetzlaff, Mathias Smialkowski, Daniel Siegmund, Jun Li, Rui Cao, and Ulf-Peter Apfel

Supplementary Information

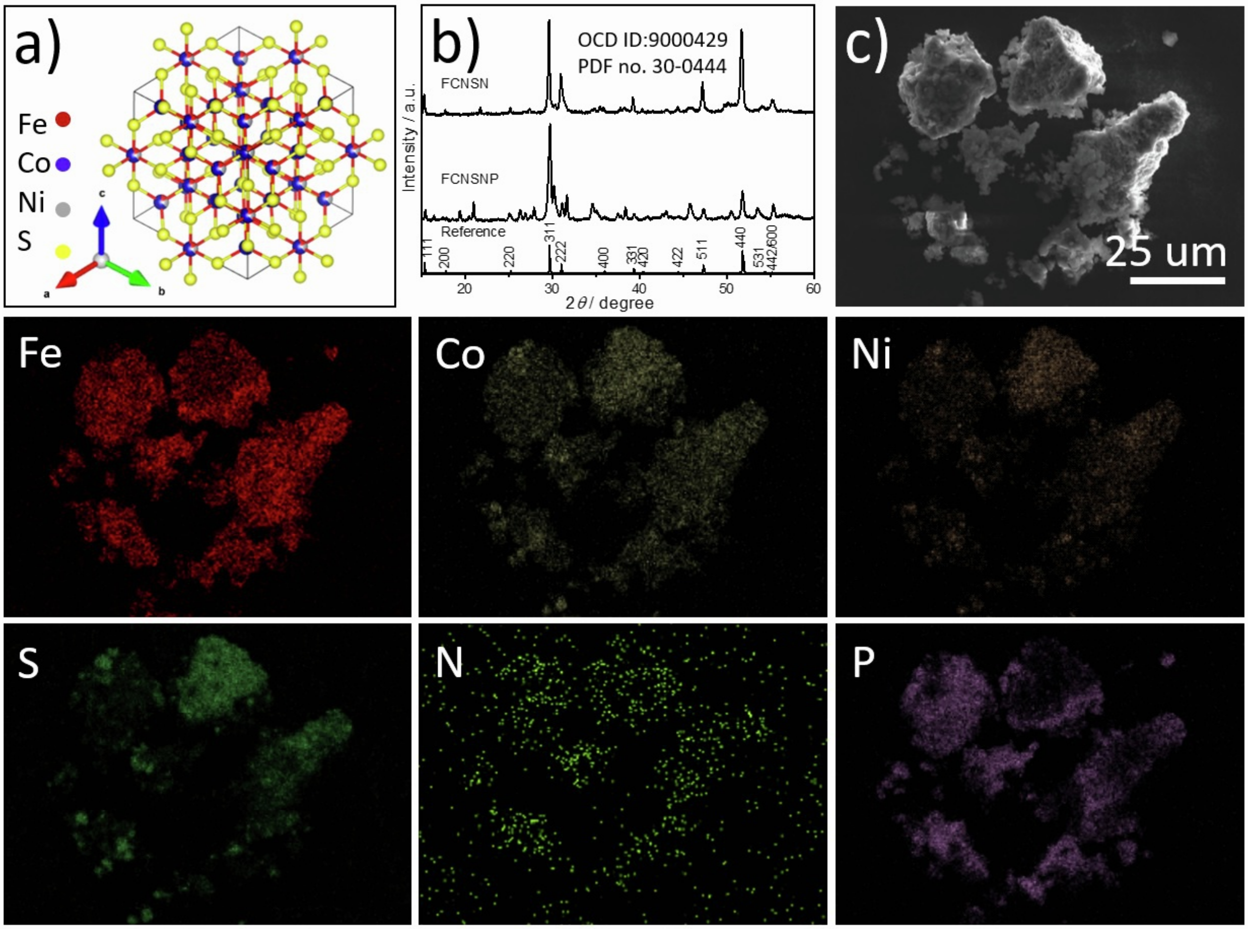

Figure S1 The crystal structure illustration of pristine trimetallic pentlandite-phase, XRD patterns of the doped samples, and SEM of FCNSNP material accompanied by atoms (Fe, Co, Ni, S, N, and P) distribution images, related to STAR Methods. a) Crystal structure illustration of trimetallic pentlandites. B) XRD patterns of FCNSNP and FCNSN samples. c) SEM image of the as-prepared FCNSNP sample and the corresponding elemental mapping images.

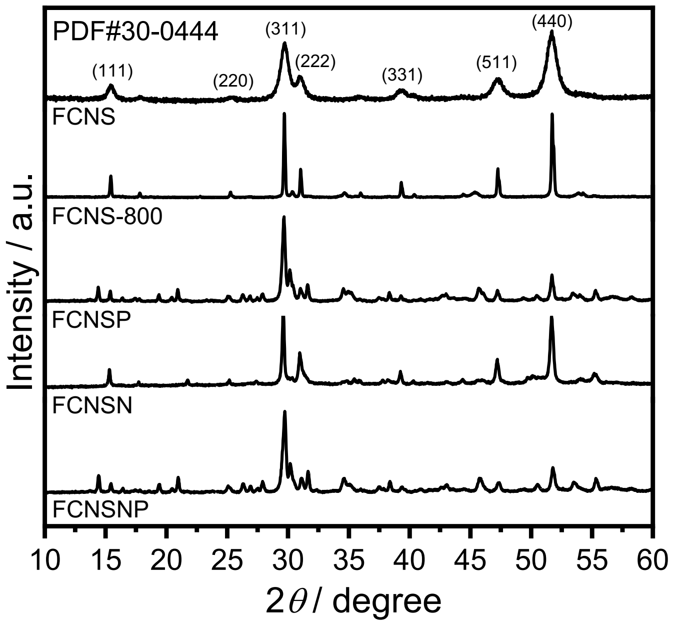

Figure S2 Wide-angle XRD patterns of the as-prepared powders, related to STAR Methods.

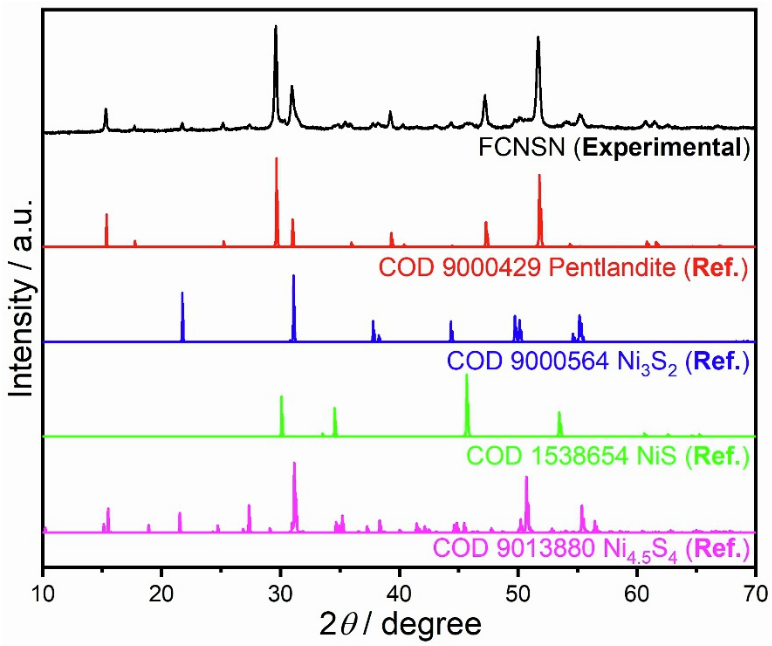

Figure S3 Phase analysis of the wide-angle XRD patterns collected on the as-prepared FCNSN powder, related to Figure 1.

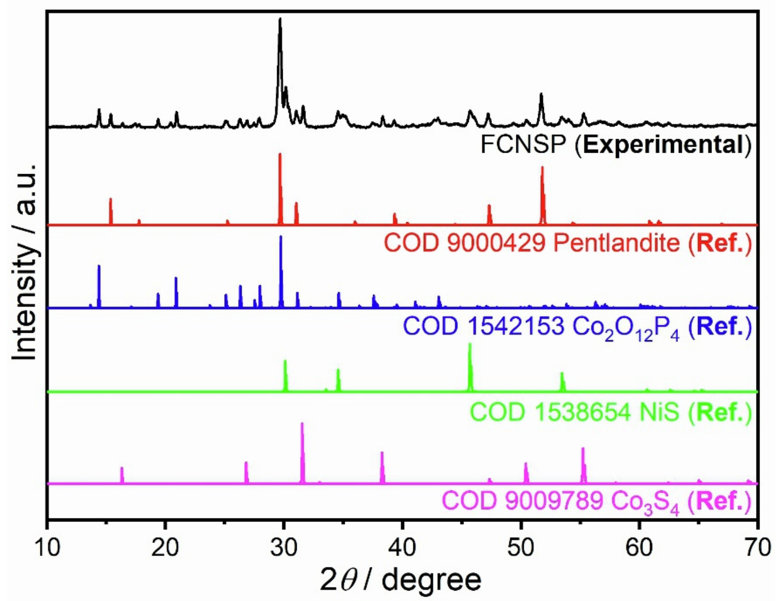

Figure S4 Phase analysis of the wide-angle XRD patterns collected on the as-prepared FCNSP powder, related to STAR Methods.

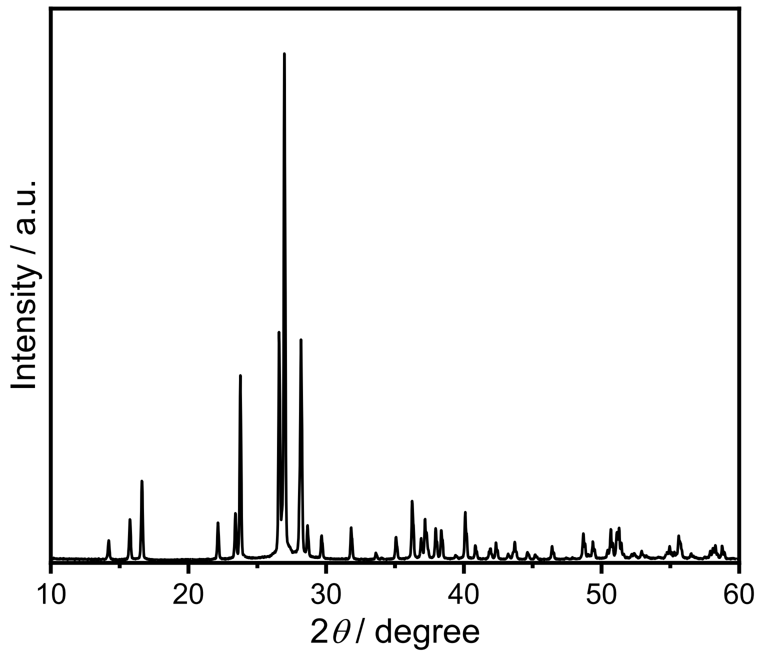

Figure S5 Wide-angle XRD patterns of cyanuric chloride, related to STAR Methods.

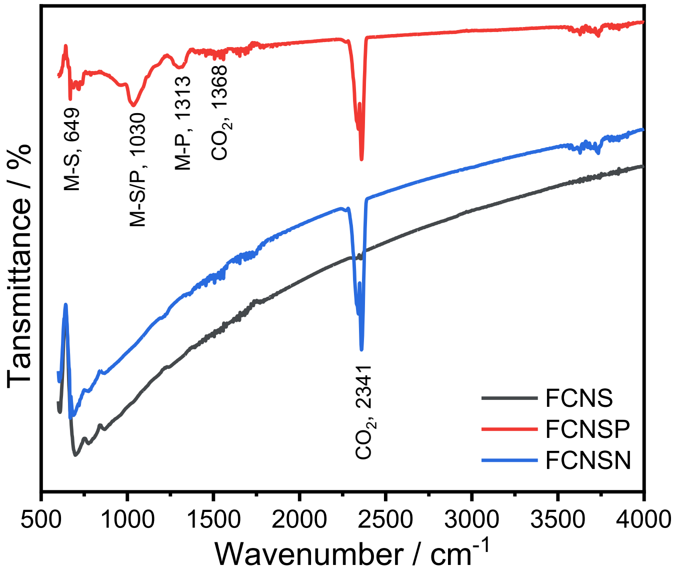

Figure S6 FTIR-ATR spectra of our materials, related to STAR Methods.

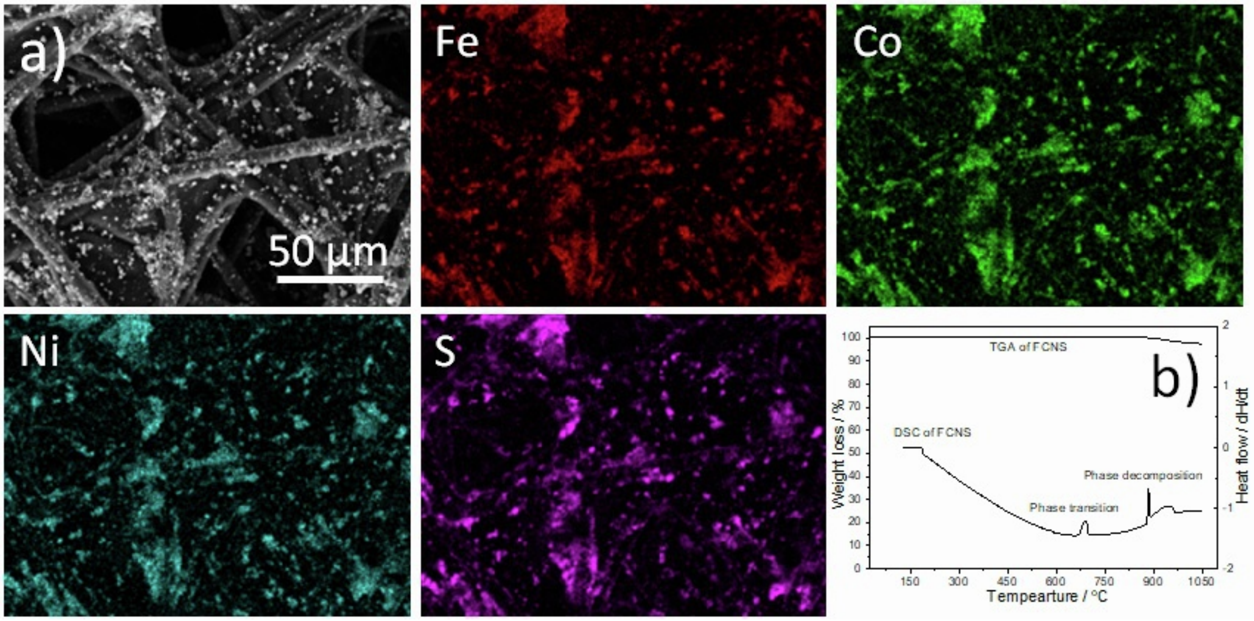

Figure S7 Top view SEM images, atoms (Fe, Co, Ni, and S) distribution images, and thermal decomposition properties of pristine FCNS, related to STAR Methods. a) SEM image of FCNS sample and the correspond elemental mapping images. b) TGA and DSC graphs of FCNS powder from room temperature up to 1050 in nitrogen with a heating rate of 5 °C min^{-1}.

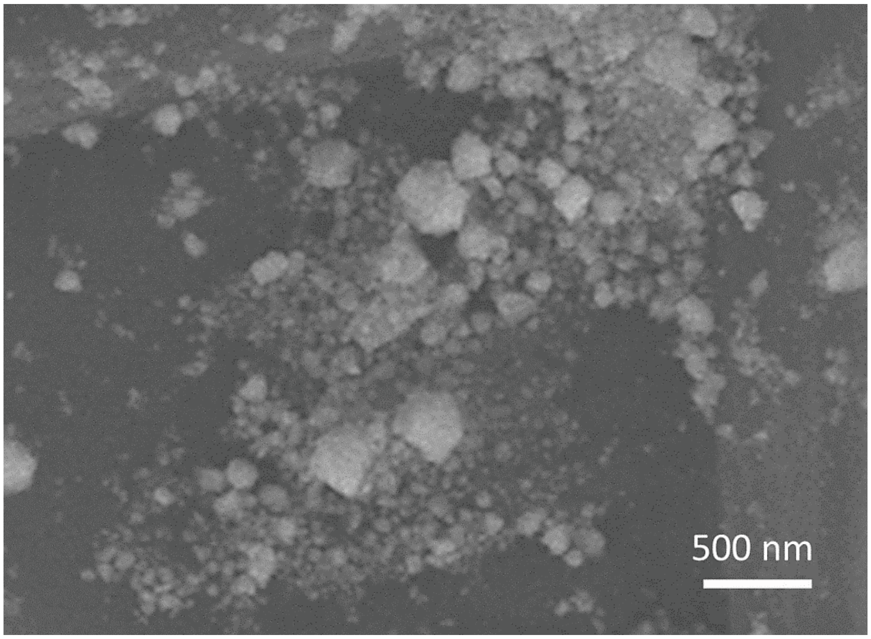

Figure S8 Top view high resolution SEM image of FCNS powder to show the average particles size, related to STAR Methods.

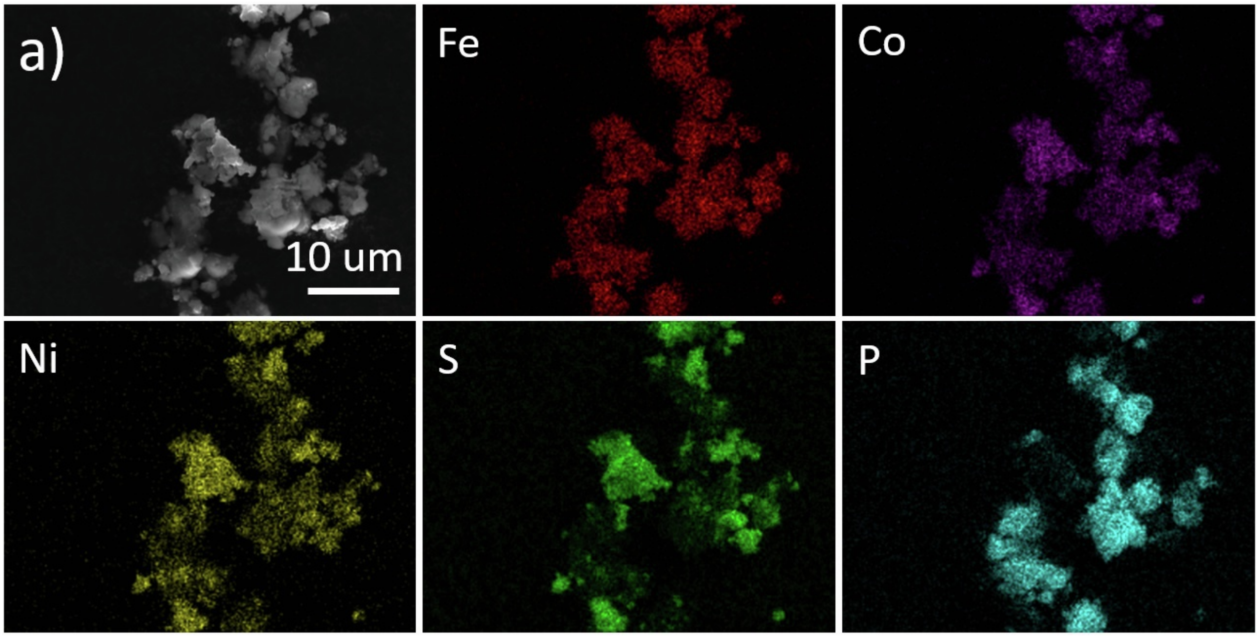

Figure S9 Top view SEM images and atoms (Fe, Co, Ni, S, and P) distribution images of FCNSP, related to STAR Methods. a) SEM of the as-prepared FCNSP sample and the correspond elemental mapping images.

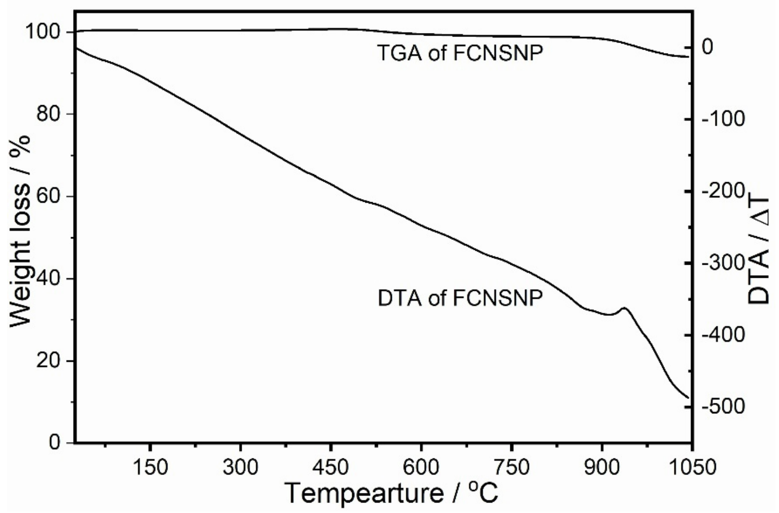

Figure S10 TGA and DSC graphs of FCNSNP powder from room temperature up to 1050 in nitrogen with a heating rate of 5 °C min^{-1}, related to Scheme 1.

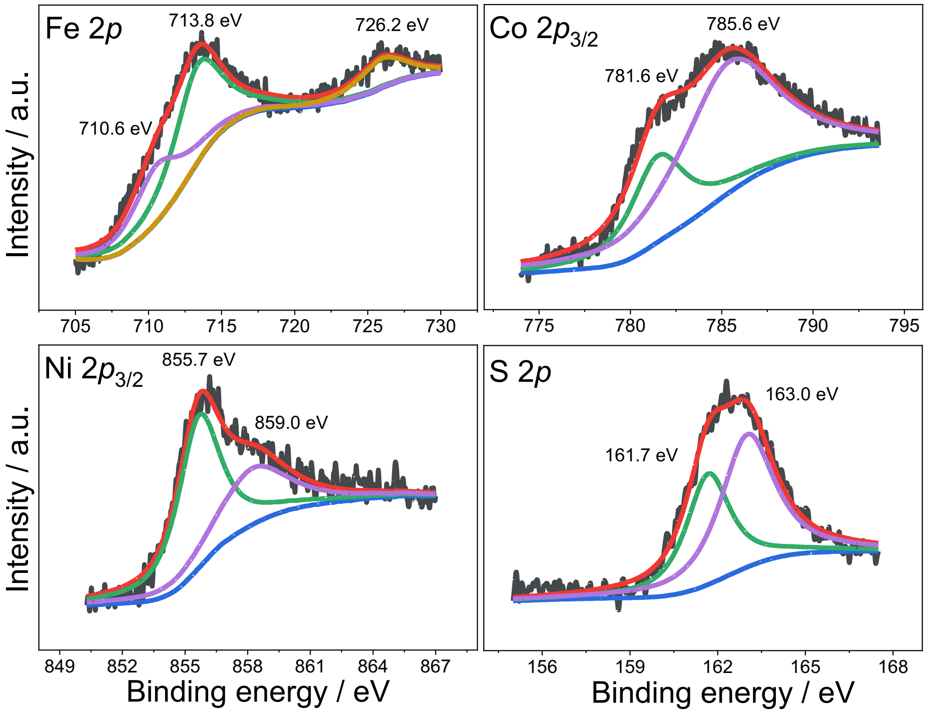

Figure S11 High-resolution XPS spectra of Fe 2*p*, Co 2*p*$_{3/2}$, Ni 2*p*$_{3/2}$, and S 2*p* orbitals collected from wide scan survey of FCNS sample, related to STAR Methods.

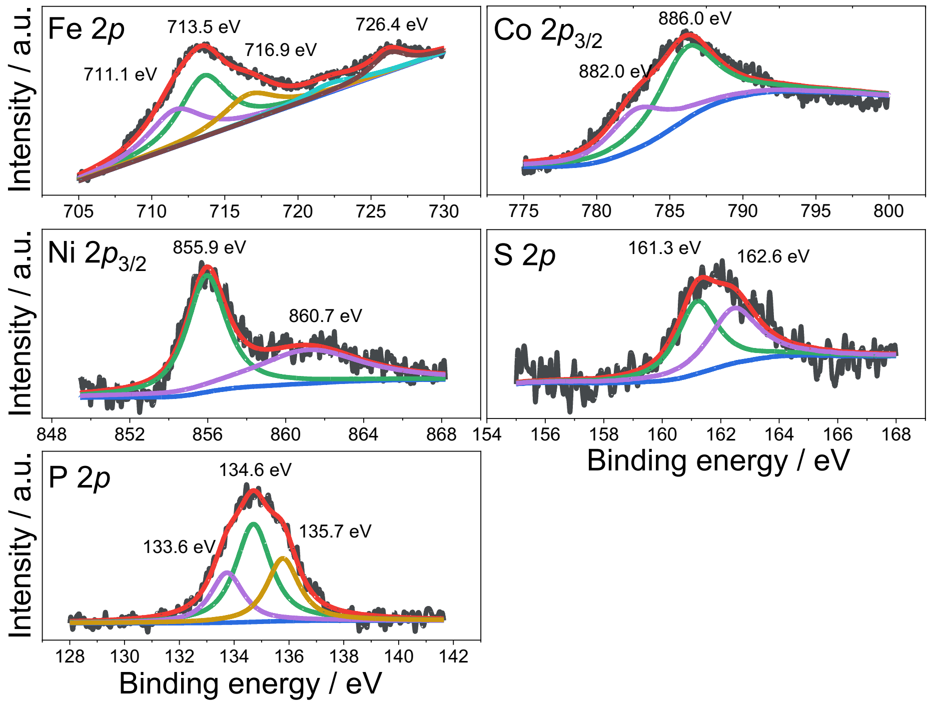

Figure S12 High-resolution XPS spectra of Fe 2*p*, Co 2*p*$_{3/2}$, Ni 2*p*$_{3/2}$, S 2*p*, and P 2*p* orbitals collected from wide scan survey of FCNSP sample, related to STAR Methods.

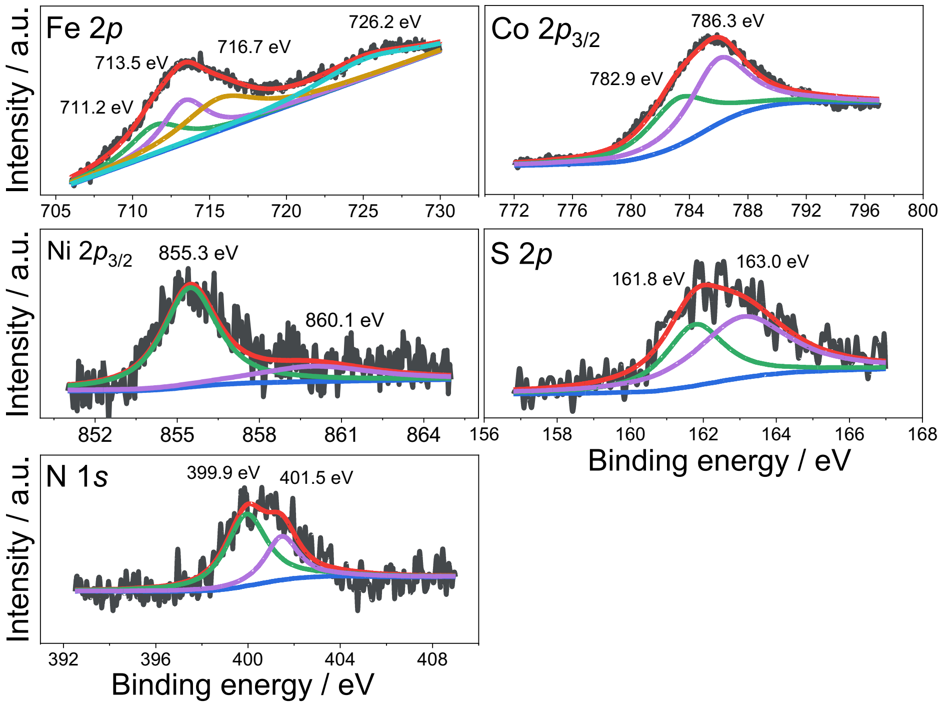

Figure S13 High-resolution XPS spectra of Fe 2*p*, Co 2*p*$_{3/2}$, Ni 2*p*$_{3/2}$, S 2*p*, and N 1*s* orbitals collected from wide scan survey of FCNSN sample, related to Figure 1.

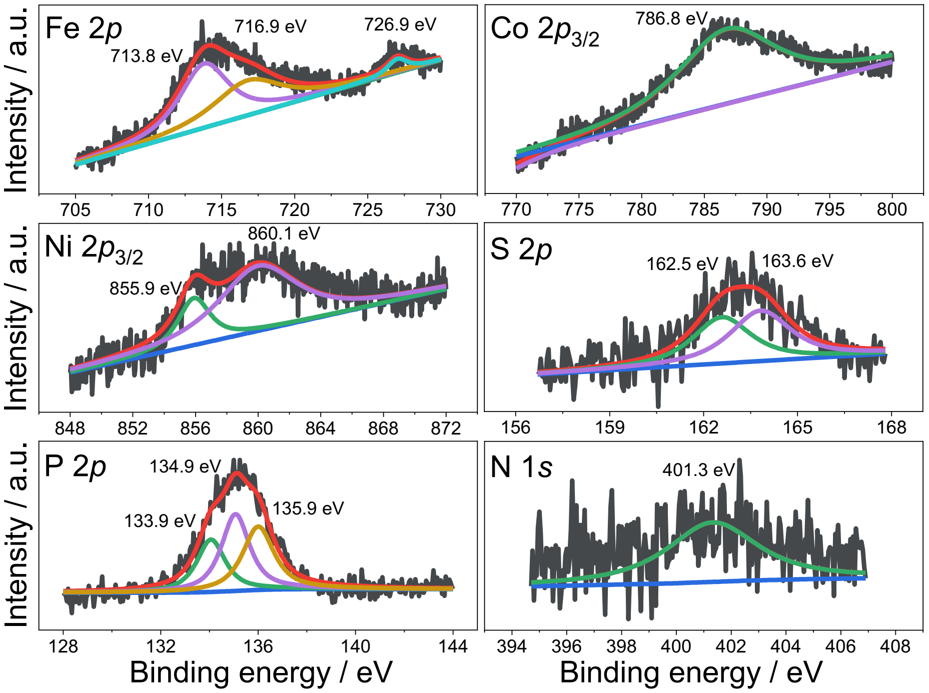

Figure S14 High-resolution XPS spectra of Fe 2*p*, Co 2*p*3/2, Ni 2*p*3/2, S 2*p*, P 2*p*, and N 1*s* orbitals collected from wide scan survey of FCNSNP sample, related to Scheme 1.

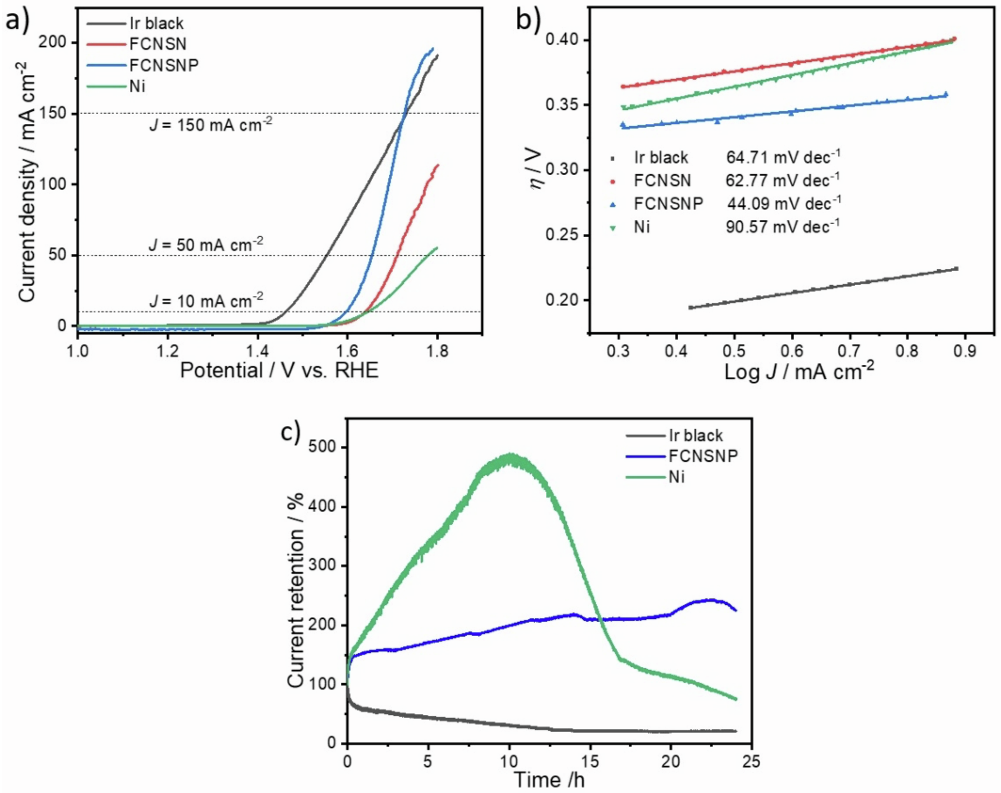

Figure S15 OER performance and stability measurements of FCNSN and FCNSNP samples, compared to the benchmarked Ir black and commercial Ni metal powder in alkaline solution, related to Figure 2. a) LSV curves of our materials in 1.0 M KOH solution at scan rate of 50 mV s^{-1} benchmarked with Ir black and Ni metal powders, b) the derived Tafel plots from LSV, and c) the long chronoamperometry test at 1.558 V vs. RHE for 24 h on GCE.

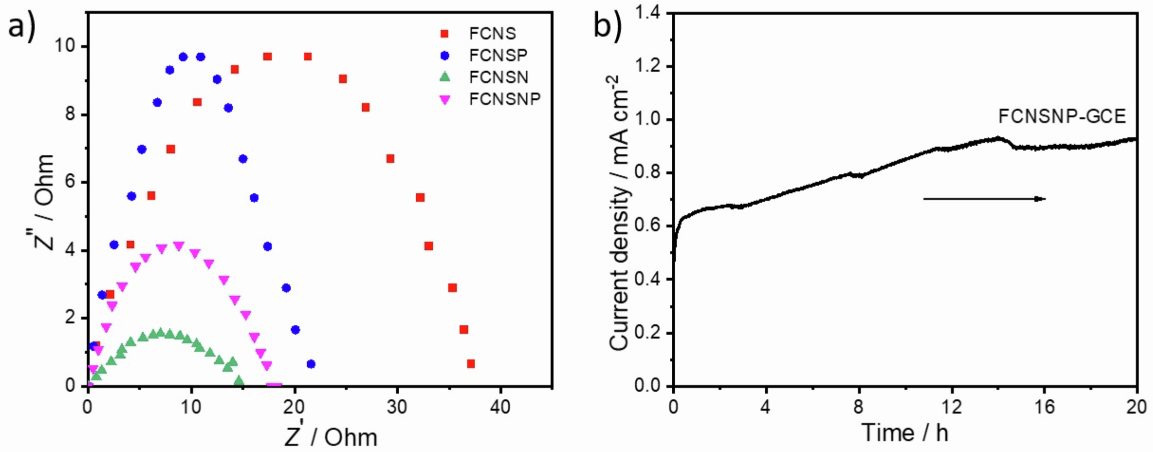

Figure S16 Charge transfer resistance measurements of FCNS, FCNSP, FCNSN, and FCNSNP samples, and stability test of FCNSNP sample in alkaline solution, related to Figure 2. a) EIS Nyquist plots recorded at 1.708 V vs. RHE in 1.0M KOH solution of all materials on GCE. b) Chronoamperometric responses collected on the FCNSNP-GCE at applied potential of 1.558 V vs. RHE for around 20 h in 1.0M KOH solution.

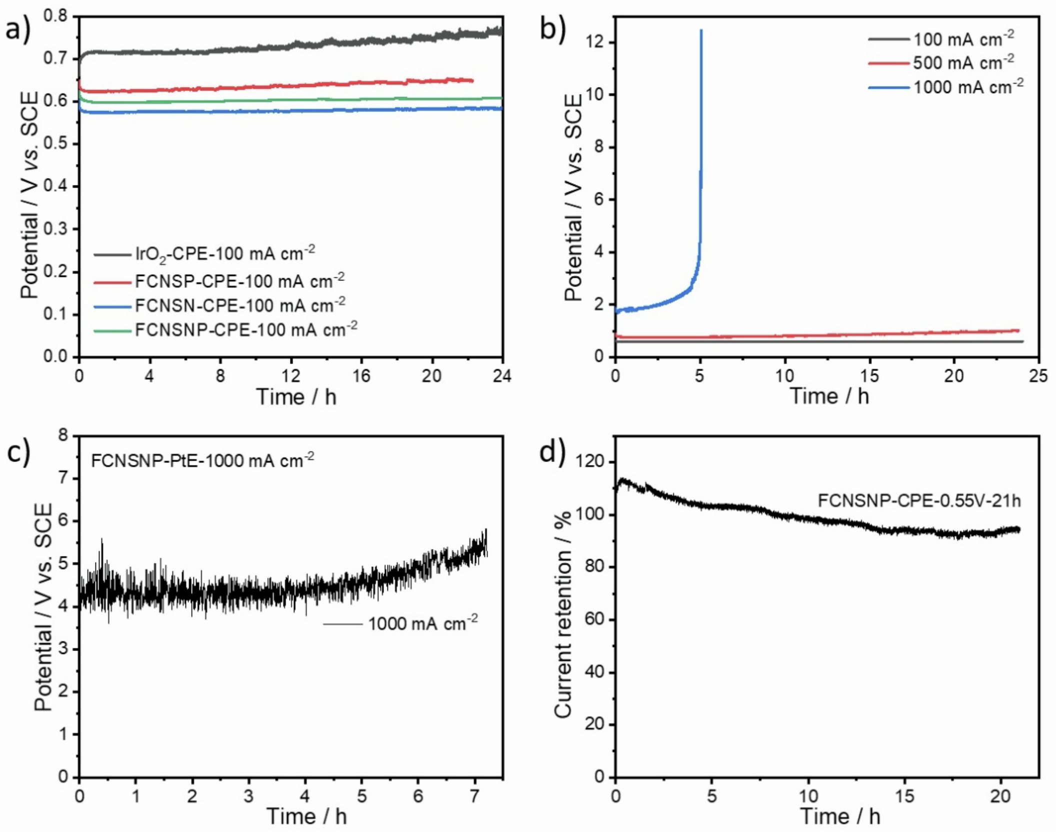

Figure S17 Long-term stability measurements of FCNSNP and FCNSNP samples at elevated current density up to 1000 mA cm^{-2} using different substrates, related to Figure 3. Long-term chronopotentiometry measurements of a) IrO_2, FCNSP, FCNSN, and FCNSNP at 100 mA cm^{-2}, and b) FCNSNP at 100, 500, and 1000 mA cm^{-2} on CPE, and c) FCNSNP at 1000 mA cm^{-2} on PtE. d) long-term chronoamperometry test of FCNSNP at 1.558 V vs. RHE on CPE

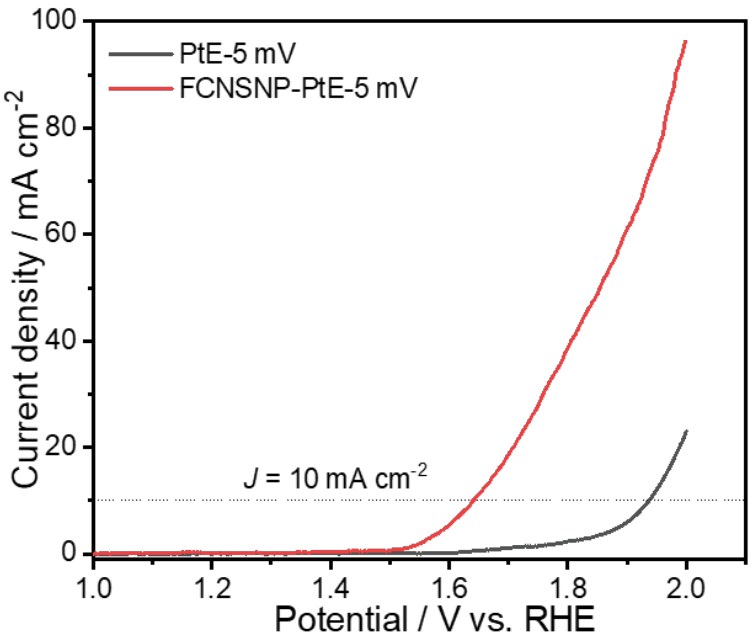

Figure S18 LSV curves of PtE and FCNSNP-PtE at 5.0 mV s^{-1} in 1.0 M KOH solution, related to Figure 3.

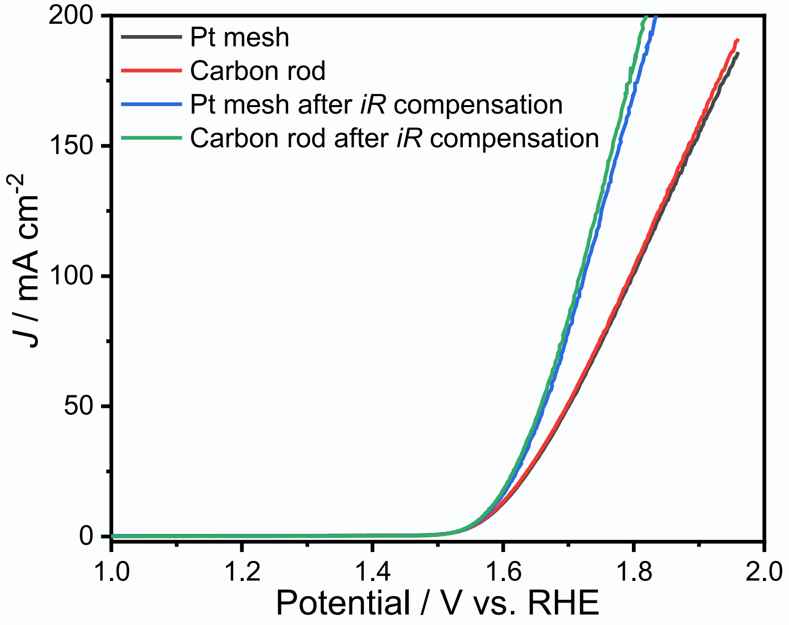

Figure S19 LSV curves using Pt and carbon counter electrode performed on FCNSNP sample, related to Figure 3.

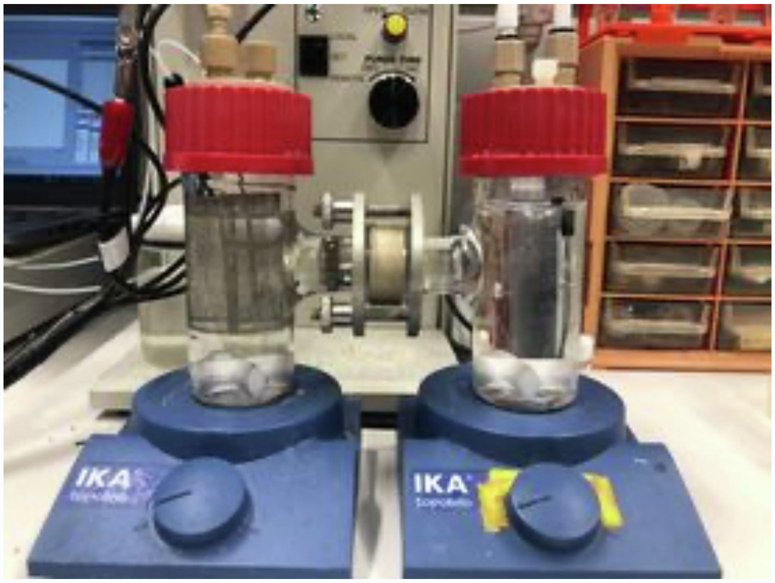

Figure S20 Our H-type cell using Pt mesh as a counter electrode, related to Figure 3.

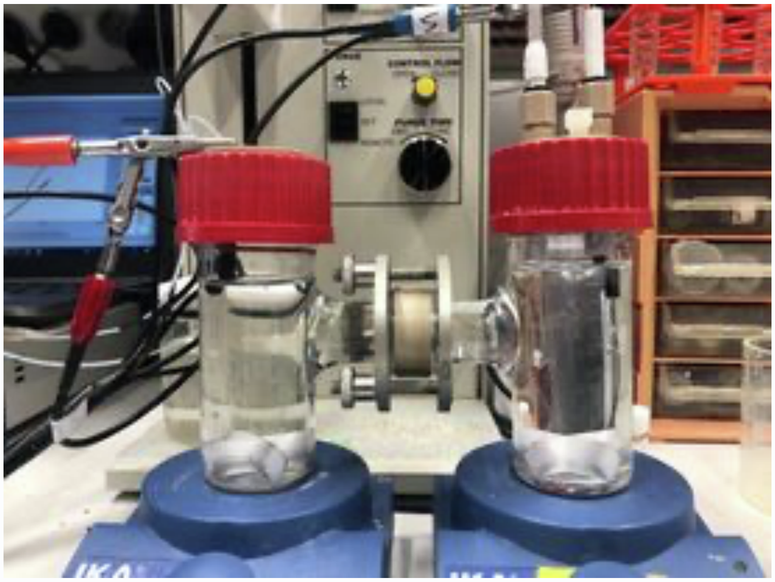

Figure S21 Our H-type cell using carbon rode as a counter electrode, related to Figure 3.

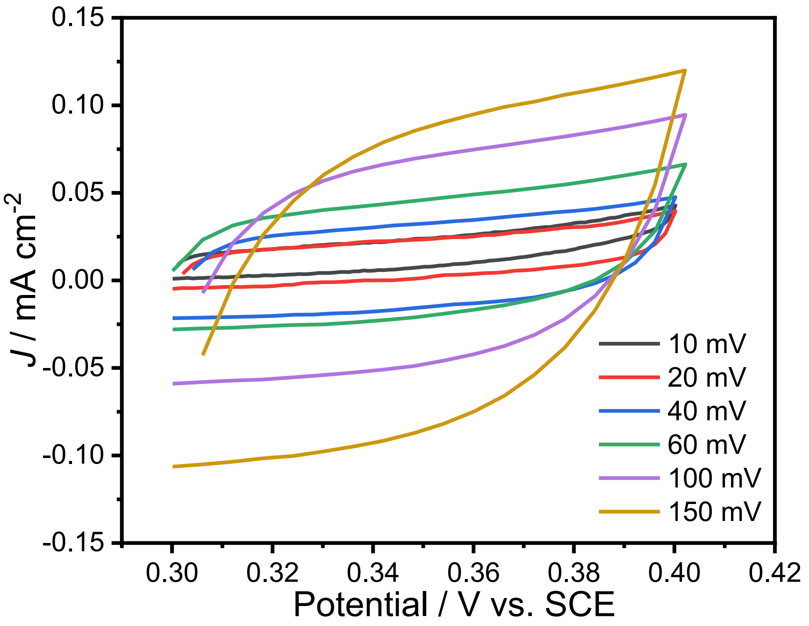

Figure S22 Cyclic voltammetry curves (CVs) of CPE recorded at 10, 20, 40, 60, 100, and 150 mV s^{-1}, related to Figure 3.

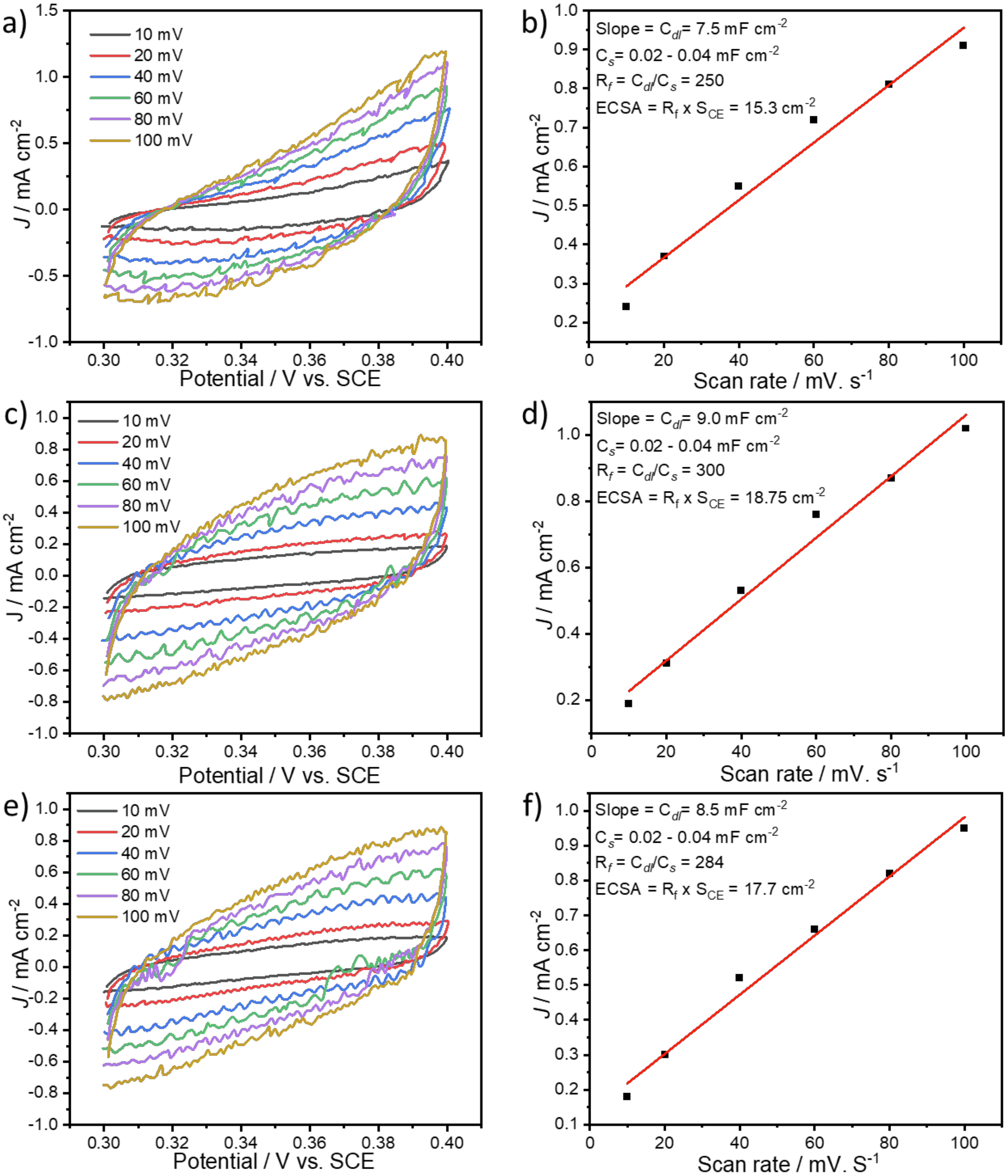

Figure S23 C_{dl} and ECSA derivation using cyclic voltammetry measurements, related to Figure 3. a,c,e) Cyclic voltammetry (CVs) curves at 10, 20, 40, 60, 80, and 100 mV s^{-1} in 1.0 M KOH solution and b,d,f) double layer charging current vs. scan rate plots of FCNSP/CPE, FCNSN/CPE, and FCNSNP/CPE, respectively.

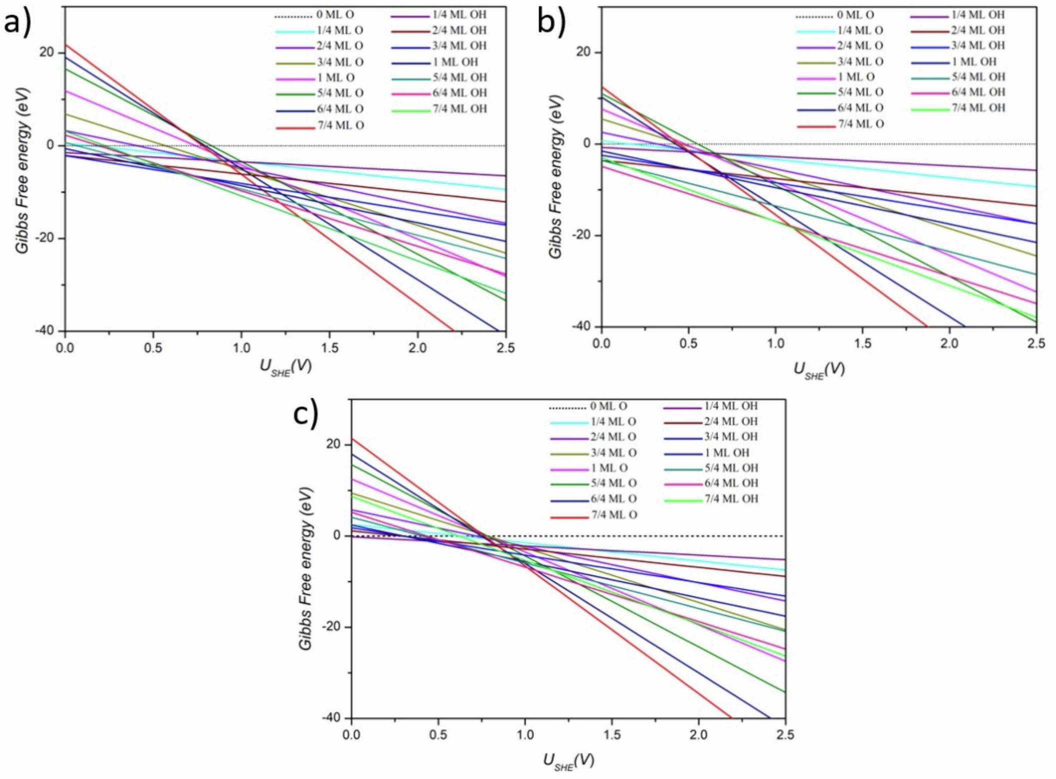

Figure S24 Comparing free energies of all coverage fraction surfaces sampled for a) FCNS, b) FCNSN and c) FCNSP at pH = 0. The surface Pourbaix diagram identifies the most stable state of the surface at given reaction conditions of potential. We predict that below ≈1.30 V (vs. RHE), FCNS surface covered by OH* is the most stable while with increase the electrode potential, the FCNS surface become covered by O*(7/4ML). In the case of FCNSN and FCNSP, the *O coverage increases to 7/4 ML at U ≈ 1.15 V and U ≈ 1.00 V vs. RHE, respectively, related to Figure 4.

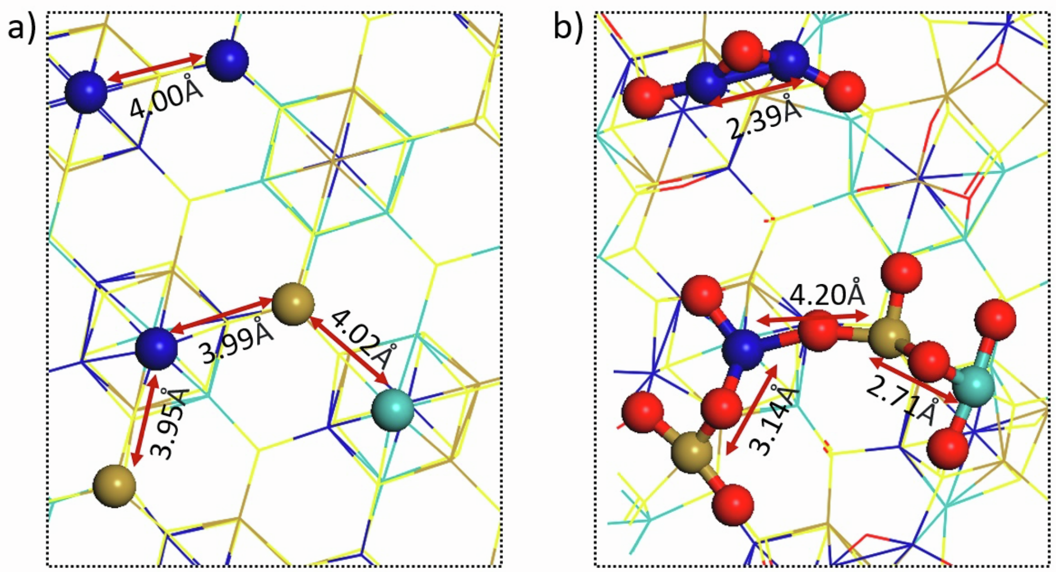

Figure S25 Structural parameters on selected metals at a) 0 O* ML coverage and b) 7/4 O* ML coverage in pristine FCNS surface, related to Figure 4.

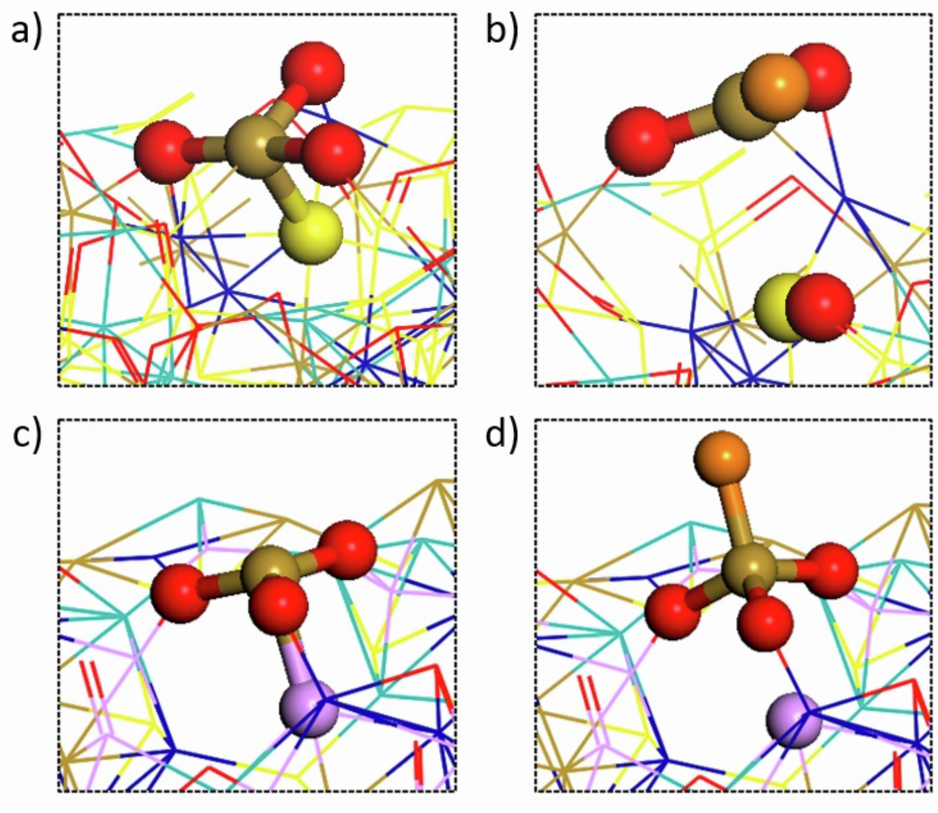

Figure S26 Dynamic surface chemistry of Fe site (in octahedral site) in oxygen evolution reaction. a) before and b) after O* adsorption in $Ni_3Fe_3Co_3S_8$ surface with 7/4 O* ML coverage. (c) before and (d) after O* adsorption in FCNSP with 7/4 O* ML coverage, related to Figure 4.

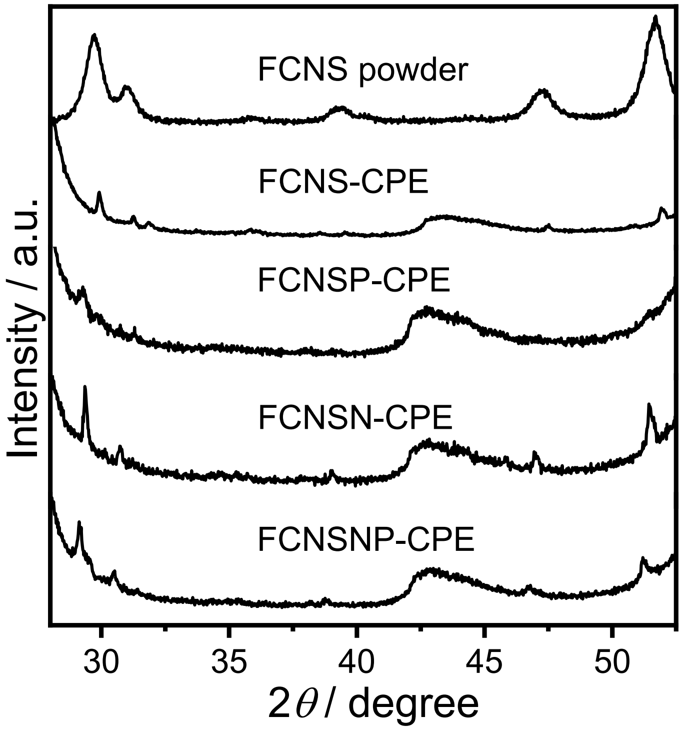

Figure S27 Wide-angle XRD patterns of the materials on carbon paper electrode (CPE), related to STAR Methods.

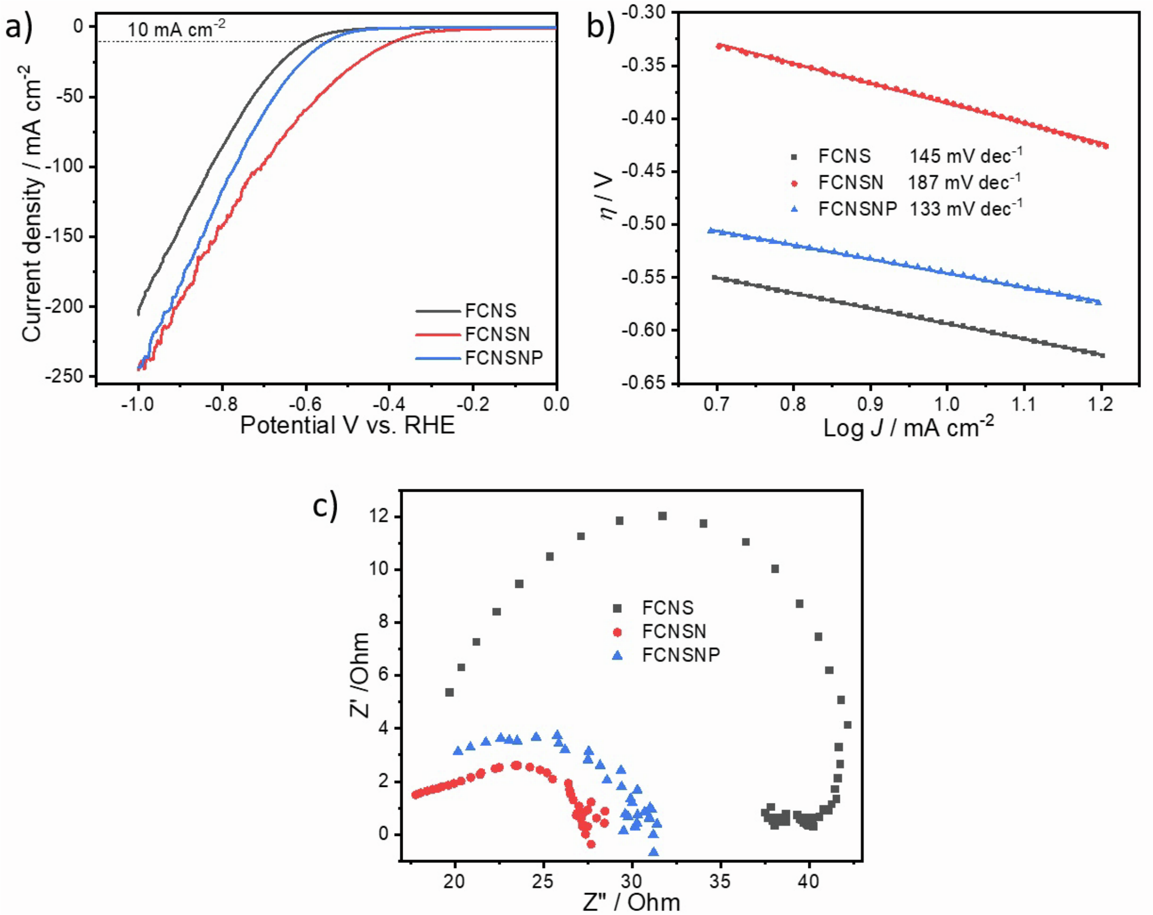

Figure S28 HER performance and stability measurements of FCNS, FCNSN, and FCNSNP samples in alkaline solution, related to Figure 6. a) LSV curves of FCNS, FCNSN, and FCNSNP samples in 1.0 M KOH solution at scan rate of 50 mV s^{-1}, b) derived Tafel plots from LSV, and e) EIS Nyquist measurements on GCE.

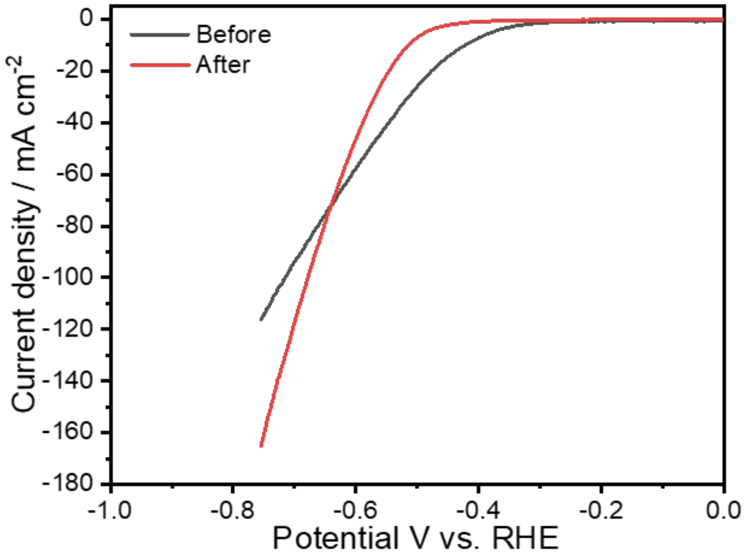

Figure S29 LSV curves of FCNSN electrode before and after chronoamperometry test at -0.355 V vs. RHE for 20 h in 0.5 M H_2SO_4 on GCE, related to Figure 6.

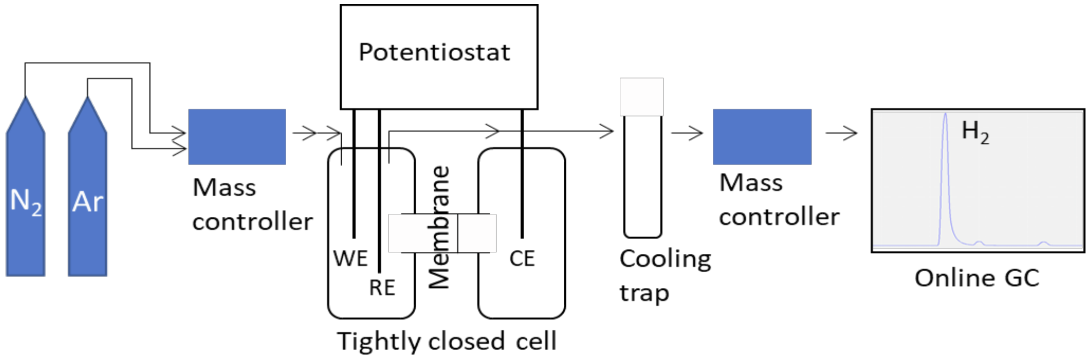

Figure S30 Schematic illustration of online gas chromatography apparatus connect to the electrochemical cell for qualitative and quantitative analysis O_2 and H_2 gases, related to Figure 7.

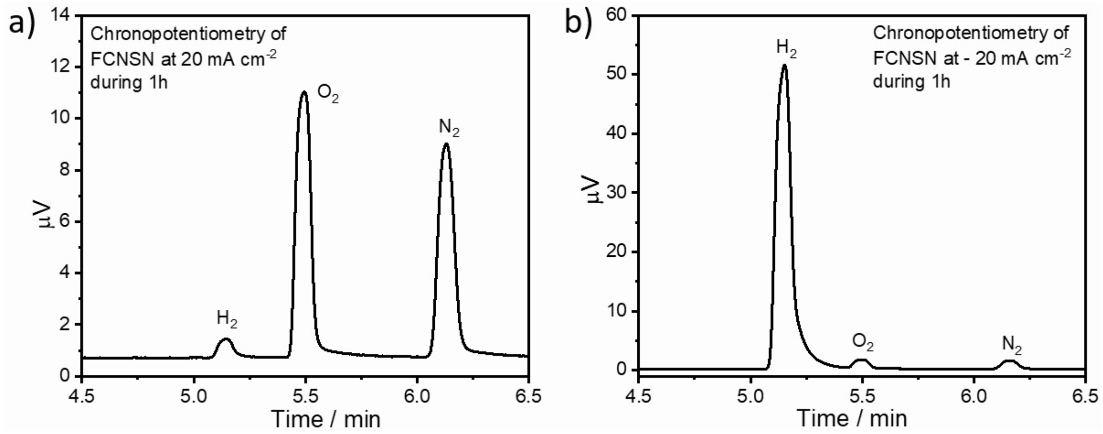

Figure S31 Gas chromatograms of a) OER at anode and b) HER at cathode during 1h at 20 mA cm^{-2} and - 20 mA cm^{-2}, respectively of FCNSN sample, related to Figure 7.

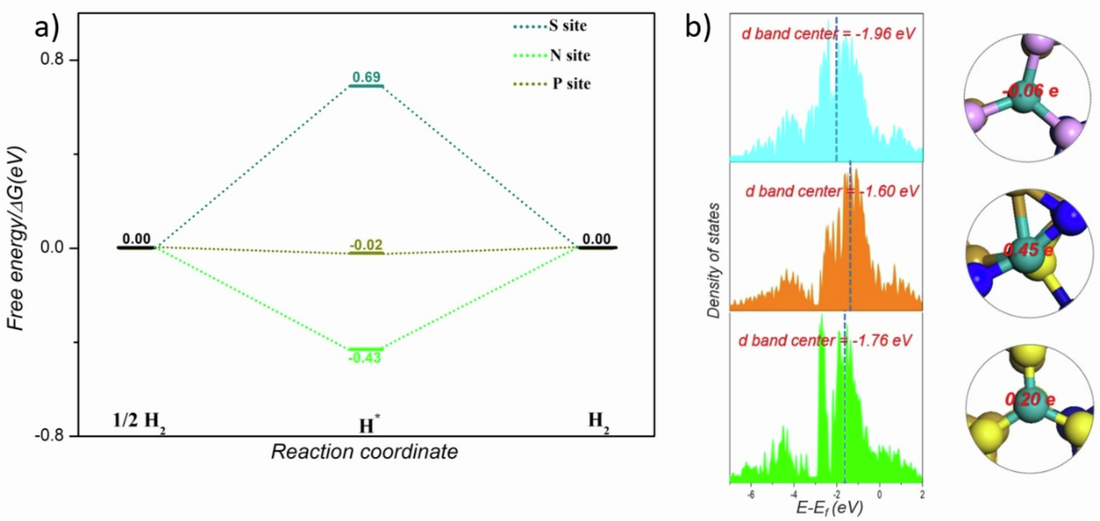

Figure S32 DFT calculation: a) Free energy diagrams of HER on S, N and P anions. b) Partial density of states (PDOS) and bader charge of Ni metal in octahedral site in pristine FCNS, FCNSN, and FCNSP, related to Figure 8.

Table S1 Atomic percentage of elements in our materials determined by ICP-OES, CHNS, and ion chromatography analyses, related to STAR Method.

Sample name	*% Fe*	*% Co*	*% Ni*	*% S*	*% N*	*% P*
FCNS	19.48	20.06	20.8	30.11	----	----
FCSNP	14.56	14.47	13.69	11.8	----	25.71
FCNSN	19.11	29.37	23.55	12.1	4.41	----

Table S2 OER performance of our materials against the previously reported pentlandites electrocatalysts, related to Figures 2 and 3. The highlighted materials are from this study.

Sample name	*J* *mA cm^{-2}*	*η* *mV*	*Tafel slope* *mV dec^{-1}*	*Electrolyte*	*Electrode*
FCNSP	10	419	76	1.0MKOH	GCE
FCNSN	10	390	64	1.0MKOH	GCE
FCNSNP	10	349	51	1.0MKOH	GCE
FCNSP	100	479	51	1.0MKOH	CPE
FCNSN	100	440	70	1.0MKOH	CPE
FCNSNP	100	427	46	1.0MKOH	CPE
Ni_9S_8	10	354	56	1.0MKOH	GCE
$FeNi_8S_8$	10	371	55	1.0MKOH	GCE
$Fe_2Ni_7S_8$	10	359	55	1.0MKOH	GCE
$Fe_3Ni_6S_8$	10	367	61	1.0MKOH	GCE
$Fe_4Ni_5S_8$	10	386	56	1.0MKOH	GCE
$Fe_5Ni_4S_8$	10	401	60	1.0MKOH	GCE
$Fe_6Ni_3S_8$	10	423	75	1.0MKOH	GCE
$Fe_7Ni_2S_8$	10	434	91	1.0MKOH	GCE
Fe_8NiS_8	10	495	75	1.0MKOH	GCE
Fe_9S_8	---	---	168	1.0MKOH	GCE
Co_9S_8 spheres	10	285	58	1.0MKOH	GCE
Co_9S_8 flowers	10	380	76	1.0MKOH	GCE
NSC/$Ni_4Fe_5S_8$-1000	10	620	431	1.0MKOH	GCE
PNSC/$Ni_4Fe_5S_8$-1000	10	300	72	0.5MH_2SO_4	GCE
PNSC/$Ni_4Fe_5S_8$-1000	10	280	---	1.0MKOH	GCE
$Ni_{4.3}Co_{4.7}S_8$	20	133	194	1.0MKOH	Ni foam

Co_9S_8/CNS	10	294	50.1	1.0MKOH	RDE
Co_9S_8	10	340	85.6	1.0MKOH	RDE

Table S3 FE% of our materials against the previously reported pentlandites electrocatalysts for HER, related to Figure 7. The highlighted materials are from this study.

Sample name	FE%	Time/h	*Electrolyte*	*Electrode*
FCNS	95.4±2	10.0	0.5 M H_2SO_4	CE
FCNSN	98.1±2	10.0	0.5 M H_2SO_4	CPE
FCNSNP	97.5±2	10.0	0.5 M H_2SO_4	CPE
$Ni_{4.5}Fe_{4.5}S_8$	91.0±5	5.0	0.5 M H_2SO_4	Rocks
$Fe_{4.5}Ni_{4.5}S_8$	90.2±5	4.0	0.5 M H_2SO_4	Pellet
$Fe_{4.5}Ni_{4.7}S_7Se_1$	94.1±5	4.0	0.5 M H_2SO_4	Pellet
$Fe_{4.5}Ni_{4.7}S_6Se_2$	98.5±5	4.0	0.5 M H_2SO_4	Pellet
$Fe_{4.5}Ni_{4.7}S_5Se_3$	97.8±5	4.0	0.5 M H_2SO_4	Pellet
$Fe_{4.5}Ni_{4.7}S_4Se_4$	95.9±5	4.0	0.5 M H_2SO_4	Pellet
$Fe_{4.5}Ni_{4.7}S_3Se_5$	96.7±5	4.0	0.5 M H_2SO_4	Pellet
